# Impact of Travel Burden on Timeliness of Care and Overall Survival for Breast Cancer: A National Cancer Database Analysis

**DOI:** 10.1002/cam4.71354

**Published:** 2025-11-10

**Authors:** Yongzhe Wang, Christine M. Quinones, Elizabeth Gonzalez, Preeti Farmah, Hans F. Schoellhammer, Lorena Gonzalez, Nikita Shah, Katharine Schulz‐Costello, Jennifer Tseng, Veronica C. Jones

**Affiliations:** ^1^ Department of Surgery City of Hope Duarte California USA; ^2^ School of Medicine University of California, Riverside Riverside California USA

**Keywords:** breast cancer, overall survival, surgery, timeliness of care, travel burden

## Abstract

**Introduction:**

Timely treatment initiation is critical to clinical outcomes in breast cancer (BC). While social determinants of health are established drivers of disparities in the timeliness of care (ToC), the impact of travel burden remains less defined. This study evaluates associations between travel burden, ToC, and overall survival (OS) in a nationally representative BC cohort.

**Methods:**

We analyzed 283,166 bc patients from the National Cancer Database (2004–2021) with documented great circle distance (GCD), categorized as ≤ 10, 10.1–20.0, and > 20 miles. Associations between GCD and ToC—defined as time from diagnosis to first treatment and categorized as < 8, 8–12, and > 12 weeks—were assessed using negative binomial models, and associations with OS were evaluated using Cox models.

**Results:**

Compared to patients with GCD < 10 miles, patients with GCD > 20 miles experienced delays in ToC when diagnosed with early‐stage disease, especially when undergoing surgery as first treatment (RR: 1.05, 95% CI: 1.04–1.06). Compared to treatment within 8 weeks of diagnosis, treatment initiation > 12 weeks had 24% higher mortality (HR: 1.24, 95% CI: 1.14–1.35), especially among patients receiving surgery (HR: 1.31, 95% CI: 1.19–1.43) and chemotherapy (HR: 1.30, 95% CI: 1.18–1.43). Even surgery within 8–12 weeks carried an elevated risk (HR: 1.09, 95% CI: 1.02–1.16). Metropolitan patients with GCD > 20 miles had a 12% lower mortality (HR: 0.88, 95% CI: 0.81–0.96) than those ≤ 10 miles away; no such differences were observed in urban or rural groups.

**Conclusion:**

Travel burden influences BC ToC and OS through geographic, clinical, and facility factors, underscoring the need for tailored interventions that address local care capacity, patient demographics, and disease profiles.

## 1 Introduction

In the treatment of breast cancer (BC), timely access to diagnostic and therapeutic interventions has been shown to significantly improve outcomes, including overall survival (OS), distinguishing BC from other cancers [1, 2]. Many factors, including health insurance status [2, 3, 4, 5, 6, 7, 8], race and ethnicity [2, 3, 4, 5, 6, 7, 8, 9, 10, 11, 12, 13, 14, 15], household income [2, 4, 5, 8, 9, 13, 16, 17, 18], and educational attainment [4, 5, 8, 9] substantially influence disparities in the timeliness of care (ToC) and survival among patients with BC.

Compared with well‐characterized social determinants of health (SDoH), the impact of geographic travel burden—typically measured as travel distance or travel time from a patient's residence to the treating facility—remains less understood. Findings from recent systematic reviews have been inconsistent [19, 20, 21, 22, 23], with effects varying across geographic contexts (e.g., metropolitan vs. rural/urban areas) and institutional settings (e.g., high‐volume centers vs. low‐resource facilities), suggesting a complex interplay between location, accessibility, and outcomes [5, 11, 12, 13, 24, 25, 26].

Prior studies have primarily examined how travel burden influences treatment selection and adherence or how SDoH shape geographic access to cancer care [5, 6, 7, 12, 16, 25, 26, 27, 28, 29]. However, few have directly evaluated the relationship between travel distance and both ToC and OS, particularly using recent, nationally representative data. This study aims to address that gap by assessing whether travel distance is associated with delays in initiating treatment and whether these delays, in turn, are linked to OS. Using data from the National Cancer Database (NCDB), which captures approximately 70% of newly diagnosed cancer cases in the United States from 2004 to 2021 [30], we evaluated these associations in a large national cohort of BC patients, with stratified analyses across key clinical and sociodemographic groups.

## 2 Methods

This study used the NCDB, a national oncology registry jointly maintained by the Commission on Cancer (CoC) of the American College of Surgeons and the American Cancer Society [30]. The NCDB captures data on patients with invasive and noninvasive cancers treated at over 1500 CoC‐affiliated facilities, including demographics, clinical characteristics, treatment modalities (surgery, chemotherapy, radiation, endocrine therapy), survival outcomes, and SDoH. We extracted data from 2004 to 2021 for patients with known or unknown breast cancer stages and documented great circle distance (GCD), representing the straight‐line distance between a patient's residence and treating facility. Given that the dataset consists of deidentified patient information, the study was deemed exempt from institutional review board oversight.

### 2.1 Outcomes and Covariates

We assessed the association between GCD, ToC, and OS. ToC was defined as the number of days from diagnosis to first definitive treatment, including surgery, chemotherapy, radiation, or endocrine therapy (Table S1). OS was based on survival status at last contact and total survival time (months) from diagnosis to follow‐up. We included covariates to adjust for potential confounders: Demographic variables (age, race and ethnicity, living area, education, income, insurance status), clinical variables (Charlson‐Deyo comorbidity index, tumor grade, cancer stage, molecular subtype), and facility characteristics (facility type and location). Living area was defined using the USDA Rural–Urban Continuum Codes, which classify counties by population size, degree of urbanization, and adjacency to metropolitan areas; NCDB collapses these into three categories: metropolitan (metro), urban, and rural. Zip code–level educational attainment was defined as the percentage of adults without a high school diploma. Median household income was also derived from the 2020 American Community Survey, adjusted for 2020 inflation, and categorized into quartiles based on the distribution of U.S. zip codes. Molecular subtype classification was available only for cases diagnosed from 2018 onward, as earlier NCDB records did not routinely capture molecular markers; cases diagnosed before 2018 were classified as unknown subtype.

### 2.2 Data Analysis

To facilitate interpretation, GCD was categorized into three groups: ≤ 10 miles, 10.1–20.0 miles, and > 20 miles. Descriptive statistics were summarized in tabular form, with stratified analyses by GCD. Chi‐squared tests were used to assess differences across groups. Multiple visualizations were created, including temporal trends in patient volume and reporting facilities. The statistical analysis was conducted in two stages. First, we examined the association between GCD and ToC. Second, we assessed the relationship between GCD, ToC, and OS.

For the ToC analysis, we used Poisson regression models estimated via generalized estimating equations (GEE) with a log link [31, 32]. GEE is a quasi‐likelihood approach that provides population‐average (marginal) estimates without requiring distributional assumptions for the error term. Robust (sandwich‐based) standard errors and an exchangeable working correlation structure were specified to account for clustering of patients within treating facilities [33]. Rate ratios were estimated to quantify relative differences in the speed at which patients received primary treatment after diagnosis across categories of GCD. The modeling approach involved (1) establishing models for the overall population using all samples and (2) conducting stratified analyses within subgroups defined by cancer stage, primary treatment, molecular subtype, facility type, and living area. All models were adjusted for demographic, socioeconomic, clinical, and facility‐level covariates. When a variable served as the stratification factor for a given analysis (e.g., molecular subtype, cancer stage, or facility type), it was not included as a covariate in the corresponding regression model. In all other analyses, that variable was in the model for adjustment. Marginal effects of GCD on the ToC outcome in days were estimated using regression models, calculated as the predicted difference in days between a given GCD category and GCD ≤ 10 miles, while holding other variables constant.

For the OS analysis, Kaplan–Meier estimates with log‐rank tests were used to compare survival distributions across categories of GCD, primary treatment, cancer stage, living area, molecular subtype, and facility type. Cox proportional hazards models were fitted to assess the association among ToC, GCD, and survival outcomes, first in the overall population and then within stratified subgroups—cancer stage, molecular subtype, living area, and facility type. Robust standard errors clustered at the facility level were used to account for within‐facility correlation [34]. For the ToC in survival analysis, prior studies in breast cancer have commonly used thresholds around 8 weeks (e.g., ≤ 8 weeks vs. > 8 weeks) in assessing delays in treatment initiation and found meaningful survival associations [35, 36, 37, 38]. We extended this by further subdividing into 8–12 weeks and > 12 weeks to allow greater granularity, while retaining interpretability to clinicians. For each subgroup (e.g., cancer stage), separate Cox models were conducted within each subset to assess subgroup‐specific associations, following the same covariate adjustment strategy described above. To ensure robustness, we conducted sensitivity analyses by varying GCD classifications. Analyses were repeated using (1) continuous GCD as a linear predictor, (2) alternative categories (≤ 5 miles, 5.1–15.0 miles, > 15 miles), and (3) an additional threshold (≤ 15 miles, 15.1–25.0 miles, > 25 miles). All ToC and OS analyses were reconducted under these specifications. Statistical significance was set at a *p* < 0.05, and all analyses were conducted using R version 4.1.1.

## 3 Results

### 3.1 Demographic and Clinical Characteristics

**FIGURE 1:**
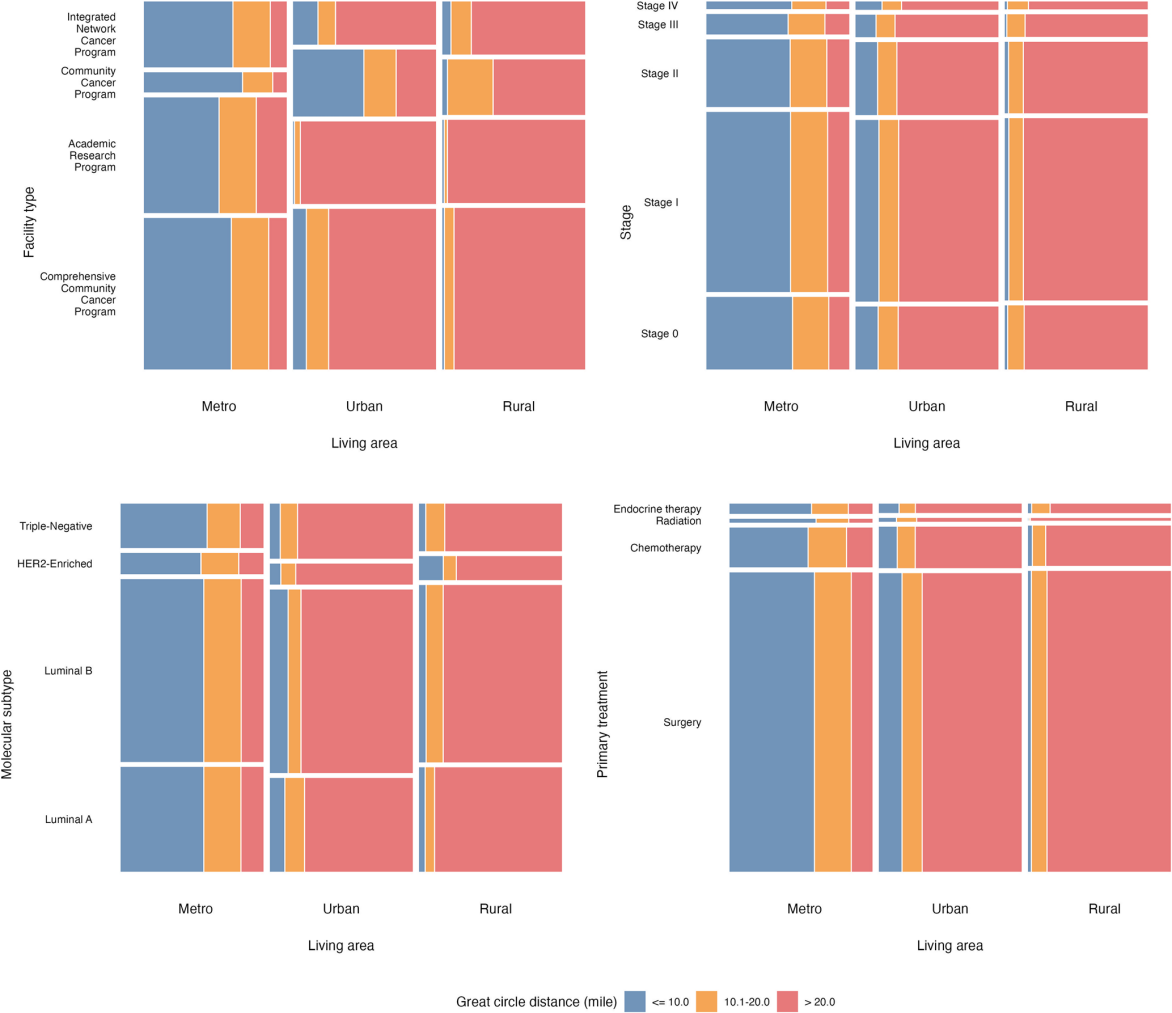
Percent stacked bar plots of great circle distance by different covariates. Plots from top left to bottom right show great circle distance by (1) living area and facility type, (2) living area and cancer stage, (3) living area and molecular subtype, and (4) living area and primary treatment. Blue, orange, and red colors represent great circle distance ≤ 10, 10.1–20, and > 20 miles.

A total of 283,166 patients were included. Of these, 53% (150,036) lived ≤ 10 miles, 23% (66,506) lived 10.1–20.0 miles, and 24% (66,624) lived > 20 miles from their treating facility. Demographic and clinical characteristics differed significantly across distance groups (Table 1). Most patients were non‐Hispanic White (82%), lived in metropolitan areas (85%), had stage I disease (50%), a Charlson–Deyo index of 0 (83%), and received definitive surgery (82%), with private insurance as the most common coverage (53%). Educational attainment, measured by area‐level percentage without a high school diploma, was significantly associated with distance (*p* < 0.001). A greater proportion of patients > 20 miles away received care at academic/research programs (ARP) (41%) compared with those ≤ 10 miles (28%). Patients living > 20 miles were more likely to reside in urban (34%) or rural (5%) areas versus those ≤ 10 miles (3% and 0.1%, respectively). Figure 1 shows comprehensive community cancer programs (CCCP) predominated in rural settings, where > 95% traveled > 20 miles, whereas most metropolitan patients traveled < 20 miles. Regardless of treatment type, subtype, or stage, > 80% of urban and rural patients traveled > 20 miles, compared with < 10% in metropolitan areas.

**TABLE 1:** Demographic and clinical characteristics stratified by great circle distance.

Characteristics	Total (*N* = 283,166, %)	Great circle distance from clinical facility to residential location in miles			*p*
		< = 10 miles (*n* = 150,036, %)	10.1–20 miles (*n* = 66,506, %)	> 20 miles (*n* = 66,624, %)	
Age at diagnosis					
40–49	49,103 (17.34%)	24,668 (16.44%)	12,464 (18.74%)	11,971 (17.97%)	< 0.001
50–59	74,589 (26.34%)	37,960 (25.3%)	18,341 (27.58%)	18,288 (27.45%)	
60–69	86,336 (30.49%)	45,384 (30.25%)	20,002 (30.08%)	20,950 (31.45%)	
70–79	55,220 (19.5%)	30,741 (20.49%)	12,194 (18.34%)	12,285 (18.44%)	
80+	17,918 (6.33%)	11,283 (7.52%)	3505 (5.27%)	3130 (4.7%)	
Zipcode‐level education: Percent no high school degree					
No HSD > = 15.3%	45,652 (16.12%)	24,209 (16.14%)	7679 (11.55%)	13,764 (20.66%)	< 0.001
No HSD 9.1%–15.2%	72,626 (25.65%)	37,194 (24.79%)	14,915 (22.43%)	20,517 (30.8%)	
No HSD 5.0%–9.0%	86,422 (30.52%)	44,357 (29.56%)	22,218 (33.41%)	19,847 (29.79%)	
No HSD < 5.0%	76,064 (26.86%)	43,052 (28.69%)	21,280 (32%)	11,732 (17.61%)	
Unknown	2402 (0.85%)	1224 (0.82%)	414 (0.62%)	764 (1.15%)	
Zipcode‐level median household income					
< $46,227	37,080 (13.09%)	20,348 (13.56%)	4321 (6.5%)	12,411 (18.63%)	< 0.001
$46,227—$57,856	53,451 (18.88%)	26,703 (17.8%)	8162 (12.27%)	18,586 (27.9%)	
$57,857—$74,062	67,436 (23.82%)	34,197 (22.79%)	16,476 (24.77%)	16,763 (25.16%)	
> = $74,063	122,146 (43.14%)	67,466 (44.97%)	36,977 (55.6%)	17,703 (26.57%)	
Unknown	3053 (1.08%)	1322 (0.88%)	570 (0.86%)	1161 (1.74%)	
Insurance					
Private Insurance	149,377 (52.75%)	76,697 (51.12%)	37,277 (56.05%)	35,403 (53.14%)	< 0.001
No	4176 (1.47%)	2181 (1.45%)	1106 (1.66%)	889 (1.33%)	
Medicaid	15,165 (5.36%)	9055 (6.04%)	2974 (4.47%)	3136 (4.71%)	
Medicare	106,867 (37.74%)	59,113 (39.4%)	23,406 (35.19%)	24,348 (36.55%)	
Other Government	2912 (1.03%)	1153 (0.77%)	791 (1.19%)	968 (1.45%)	
Unknown	4669 (1.65%)	1837 (1.22%)	952 (1.43%)	1880 (2.82%)	
Race and ethnicity					
White, NH	231,986 (81.93%)	117,119 (78.06%)	55,943 (84.12%)	58,924 (88.44%)	< 0.001
Black, NH	29,699 (10.49%)	19,682 (13.12%)	5801 (8.72%)	4216 (6.33%)	
Hispanic	4443 (1.57%)	3021 (2.01%)	837 (1.26%)	585 (0.88%)	
Asian, NH	10,545 (3.72%)	6845 (4.56%)	2476 (3.72%)	1224 (1.84%)	
AIAN, NH	925 (0.33%)	341 (0.23%)	179 (0.27%)	405 (0.61%)	
NHPI, NH	634 (0.22%)	329 (0.22%)	180 (0.27%)	125 (0.19%)	
Other	2483 (0.88%)	1428 (0.95%)	543 (0.82%)	512 (0.77%)	
Unknown	2451 (0.87%)	1271 (0.85%)	547 (0.82%)	633 (0.95%)	
Living area					
Metro	240,968 (85.1%)	144,114 (96.05%)	61,042 (91.78%)	35,812 (53.75%)	< 0.001
Urban	31,432 (11.1%)	4936 (3.29%)	4119 (6.19%)	22,377 (33.59%)	
Rural	3952 (1.4%)	74 (0.05%)	394 (0.59%)	3484 (5.23%)	
Unknown	6814 (2.41%)	912 (0.61%)	951 (1.43%)	4951 (7.43%)	
Facility type					
Comprehensive Community Cancer Program	121,650 (42.96%)	65,621 (43.74%)	29,019 (43.63%)	27,010 (40.54%)	< 0.001
Academic/Research Program	90,811 (32.07%)	42,470 (28.31%)	20,978 (31.54%)	27,363 (41.07%)	
Community Cancer Program	20,677 (7.3%)	12,754 (8.5%)	4389 (6.6%)	3534 (5.3%)	
Integrated Network Cancer Program	50,028 (17.67%)	29,191 (19.46%)	12,120 (18.22%)	8717 (13.08%)	
Facility location					
West	45,075 (15.92%)	26,101 (17.4%)	10,162 (15.28%)	8812 (13.23%)	< 0.001
South	97,169 (34.32%)	45,860 (30.57%)	24,338 (36.6%)	26,971 (40.48%)	
Midwest	82,535 (29.15%)	42,470 (28.31%)	18,009 (27.08%)	22,056 (33.11%)	
Northeast	58,387 (20.62%)	35,605 (23.73%)	13,997 (21.05%)	8785 (13.19%)	
Charlson–Deyo index					
0	236,227 (83.42%)	124,087 (82.7%)	56,044 (84.27%)	56,096 (84.2%)	< 0.001
1	34,923 (12.33%)	19,037 (12.69%)	7925 (11.92%)	7961 (11.95%)	
2	7824 (2.76%)	4452 (2.97%)	1653 (2.49%)	1719 (2.58%)	
3	4192 (1.48%)	2460 (1.64%)	884 (1.33%)	848 (1.27%)	
Tumor grade					
Grade I	58,563 (20.68%)	31,286 (20.85%)	13,560 (20.39%)	13,717 (20.59%)	< 0.001
Grade II	109,711 (38.74%)	57,867 (38.57%)	25,899 (38.94%)	25,945 (38.94%)	
Grade III	67,189 (23.73%)	35,221 (23.48%)	15,748 (23.68%)	16,220 (24.35%)	
Grade IV	1016 (0.36%)	550 (0.37%)	236 (0.35%)	230 (0.35%)	
Unknown	46,687 (16.49%)	25,112 (16.74%)	11,063 (16.63%)	10,512 (15.78%)	
Cancer stage					
Stage 0	56,235 (19.86%)	30,828 (20.55%)	13,171 (19.8%)	12,236 (18.37%)	< 0.001
Stage I	142,530 (50.33%)	75,359 (50.23%)	33,729 (50.72%)	33,442 (50.2%)	
Stage II	54,119 (19.11%)	28,165 (18.77%)	12,520 (18.83%)	13,434 (20.16%)	
Stage III	16,158 (5.71%)	8157 (5.44%)	3759 (5.65%)	4242 (6.37%)	
Stage IV	5945 (2.1%)	3167 (2.11%)	1288 (1.94%)	1490 (2.24%)	
Unknown	8179 (2.89%)	4360 (2.91%)	2039 (3.07%)	1780 (2.67%)	
Primary treatment					
Surgery	231,795 (81.86%)	123,528 (82.33%)	54,551 (82.02%)	53,716 (80.63%)	< 0.001
Chemotherapy	30,902 (10.91%)	15,019 (10.01%)	7437 (11.18%)	8446 (12.68%)	
Radiation	2745 (0.97%)	1469 (0.98%)	571 (0.86%)	705 (1.06%)	
Endocrine therapy	7546 (2.66%)	3879 (2.59%)	1753 (2.64%)	1914 (2.87%)	
Cancer subtype					
Luminal A	4054 (1.43%)	2141 (1.43%)	966 (1.45%)	947 (1.42%)	0.02
Luminal B	7196 (2.54%)	3776 (2.52%)	1682 (2.53%)	1738 (2.61%)	
HER2‐Enriched	827 (0.29%)	415 (0.28%)	195 (0.29%)	217 (0.33%)	
Triple‐Negative	1781 (0.63%)	939 (0.63%)	372 (0.56%)	470 (0.71%)	
Unknown	269,308 (95.11%)	142,765 (95.15%)	63,291 (95.17%)	63,252 (94.94%)	

**FIGURE 2:**
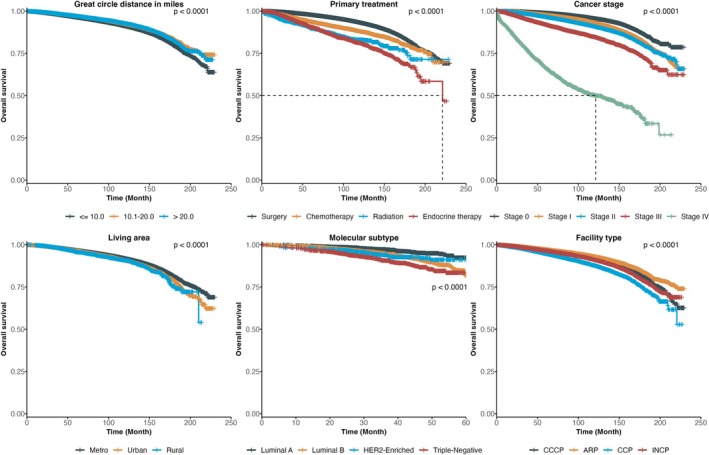
Kaplan–Meier estimates of overall survival by great circle distance, primary treatment, cancer stage, living area, molecular subtype, and facility type. Kaplan–Meier survival curves illustrating overall survival stratified by (1) great circle distance in miles, (2) primary treatment modality, (3) cancer stage, (4) living area, (5) molecular subtype, and (6) facility type (Comprehensive Community Cancer Program—CCCP, Academic/Research Program—ARP, Community Cancer Program—CCP, Integrated Network Cancer Program—INCP). Survival probabilities were estimated from the time of diagnosis and compared using the log‐rank test. Legends for each panel denote the corresponding subgroup categories, with *p* values indicating global log‐rank test significance.

Overall survival differed substantially across patient, cancer stage, and facility characteristics (Figure 2). Patients living farther from treatment facilities experienced worse survival compared with those residing within 10 miles. Survival also varied by primary treatment modality, with endocrine therapy and chemotherapy groups showing the poorest outcomes. Advancing cancer stage was strongly associated with reduced survival, particularly for stage III and IV disease. Differences were also observed by living area, with rural patients faring worse than those in metropolitan regions. Molecular subtype demonstrated inferior survival among triple‐negative and HER2‐enriched subtypes relative to luminal A and B. Finally, facility type was associated with survival, with patients treated at ARP and integrated network cancer program (INCP) centers showing less favorable outcomes compared with those treated at CCCP or community cancer program (CCP) facilities.

### 3.2 Primary Model: Full Cohort Analysis

#### 3.2.1 GCD Versus ToC

The median time from diagnosis to primary treatment was 34 days (Interquartile range [IQR]: 21–51) for patients residing ≤ 10 miles, 35 days (IQR: 22–51) for those 10.1–20.0 miles, and 35 days (IQR: 22–53) for those > 20 miles from their treating facility. In adjusted models, patients living 10.1–20.0 miles and > 20 miles experienced delays of 0.36 days (95% confidence interval [CI]: 0.01–0.70) and 1.88 days (95% CI: 1.58–2.18), respectively, compared with those within 10 miles (Table 2).

**TABLE 2:** Predicted marginal differences with 95% confidence intervals in time to treatment by great circle distance categories (compared with ≤ 10 miles).

Model type	Great circle distance in miles (REFERENCE: ≤ 10 miles)^a^	
	10.1–20.0	> 20.0
Overall	0.36* (0.01, 0.70)	1.88*** (1.58, 2.18)
Stratification: primary treatment		
Surgery	0.37* (0.02, 0.71)	2.15*** (1.83, 2.47)
Chemotherapy	0.15 (−4.91, 5.21)	0.56 (−2.96, 4.09)
Radiation	1.18 (−35.69, 38.05)	1.14 (−4.98, 7.26)
Endocrine therapy	0.76 (−1.41, 2.94)	0.63 (−1.72, 2.97)
Stratification: cancer stage		
Stage 0	0.63 (−0.31, 1.57)	3.24*** (2.17, 4.30)
Stage I	0.39 (0.20, 0.59)	1.89*** (1.64, 2.14)
Stage II	0.17 (−0.60, 0.95)	1.32** (0.53, 2.12)
Stage III	0.72 (−0.65, 2.08)	0.99 (−0.58, 2.57)
Stage IV	−4.44 (−10.05, 1.18)	−1.88 (−6.94, 3.17)
Stratification: living area		
Metro	0.46** (0.14, 0.78)	2.02** (1.71, 2.34)
Urban	−0.18 (−1.08, 0.72)	0.82 (−0.05, 1.69)
Rural	1.48 (−4.12, 7.09)	3.60 (−1.13, 8.34)
Stratification: cancer subtype		
Luminal A	1.82 (−0.67, 4.30)	2.81* (0.31, 5.31)
Luminal B	1.47 (−0.76, 3.68)	3.83*** (1.77, 5.88)
HER2‐Enriched	1.70 (−3.31, 6.71)	5.52 (−2.54, 13.58)
Triple‐Negative	0.35 (−2.40, 3.10)	2.09 (−1.05, 5.23)
Stratification: facility type		
Comprehensive Community Cancer Program	0.26 (−0.18, 0.69)	1.52*** (0.90, 2.14)
Academic/Research Program	0.68* (0.21, 1.41)	3.13*** (2.45, 3.80)
Community Cancer Program	0.31 (−0.79, 1.41)	0.17 (−1.13, 1.48)
Integrated Network Cancer Program	0.88 (−0.16, 1.92)	0.14 (−0.75, 1.02)

^a^ All values represent predicted marginal differences in days.

* < 0.05.

** < 0.01.

*** < 0.001.

#### 3.2.2 Living Area Versus ToC

Patients in urban and rural areas received treatment 5% (Rate ratio [RR]: 0.95, 95% CI: 0.94–0.96) and 10% (RR: 0.90, 95% CI: 0.87–0.92) faster, respectively, than those in metropolitan areas. Treatment at an ARP and INCP was associated with delays of 15% (RR: 1.15, 95% CI: 1.14–1.16) and 7% (RR: 1.07, 95% CI: 1.06–1.08), respectively, compared with CCCP.

#### 3.2.3 Treatment Versus ToC

The median time from diagnosis to treatment was under 45 days across all primary modalities. Endocrine therapy had the shortest median at 28 days (IQR: 16–49), followed by surgery at 35 days (IQR: 22–52). The median time from diagnosis to treatment ranged from 29 days for Stage IV to 35 days for Stage 0 and I. Compared with surgery, chemotherapy and endocrine therapy were associated with 9% (RR: 0.91; 95% CI: 0.90–0.92) and 10% (RR: 0.90; 95% CI: 0.88–0.92) faster initiation, while radiation was linked to a 27% delay (RR: 1.27; 95% CI: 1.23–1.33) (Table S2).

#### 3.2.4 ToC Versus OS

In the adjusted Cox model, treatment initiation > 12 weeks was linked to 24% higher mortality (Hazard ratio [HR]: 1.24, 95% CI: 1.14–1.35). Predicted survival was 0.06% lower at 1 year and 1.18% lower at 15 years for those treated > 12 weeks versus within 8 weeks (Figure S1), with no significant difference for those treated within 8–12 weeks. Residing > 20 miles was associated with 12% lower mortality (HR: 0.88, 95% CI: 0.81–0.96) (Table 3). Compared to surgery as first treatment, chemotherapy (HR: 1.41, 95% CI: 1.31–1.51), radiation (HR: 1.44, 95% CI: 1.24–1.68), and endocrine therapy (HR: 1.36, 95% CI: 1.24–1.49) as first treatments were associated with higher mortality (Table S3).

**TABLE 3:** Estimated hazard ratios with 95% confidence intervals from adjusted Cox models for great circle distance categories (compared with ≤ 10 miles) and time from diagnosis to initial treatment (compared with < 8 weeks).

Model type	Great circle distance in miles (Reference: ≤ 10 miles)		Weeks from diagnosis to initial treatment (reference: < 8 weeks)	
	10.1–20.0	> 20.0	8–12	> 12
Overall	0.95 (0.9, 1.01)	0.88** (0.81, 0.96)	1.04 (0.98, 1.10)	1.24*** (1.14, 1.35)
Stratification: primary treatment				
Surgery	0.96 (0.9, 1.03)	0.89* (0.81, 0.98)	1.09** (1.02, 1.16)	1.31*** (1.19, 1.43)
Chemotherapy	0.92 (0.81, 1.03)	0.86* (0.75, 0.99)	0.98 (0.85, 1.13)	1.14 (0.91, 1.42)
Radiation	1.30 (0.95, 1.79)	0.99 (0.68, 1.44)	1.04 (0.67, 1.62)	0.78 (0.50, 1.22)
Endocrine therapy	0.83 (0.66, 1.04)	0.84 (0.65, 1.08)	0.82 (0.63, 1.07)	1.32 (0.96, 1.83)
Stratification: cancer stage				
Stage 0	1.02 (0.9, 1.16)	0.91 (0.77, 1.08)	1.07 (0.94, 1.21)	1.25** (1.06, 1.47)
Stage I	0.94 (0.88, 1.02)	0.90* (0.81, 0.99)	1.13** (1.05, 1.23)	1.27*** (1.13, 1.44)
Stage II	0.94 (0.86, 1.03)	0.91 (0.81, 1.02)	1.03 (0.92, 1.15)	1.47*** (1.26, 1.70)
Stage III	1.03 (0.89, 1.19)	0.86 (0.73, 1.01)	1.03 (0.89, 1.19)	1.22 (0.99, 1.50)
Stage IV	0.93 (0.8, 1.07)	0.82* (0.69, 0.99)	0.93 (0.76, 1.13)	0.74* (0.55, 0.99)
Stratification: living area				
Metro	0.95 (0.89, 1.02)	0.88** (0.81, 0.96)	1.05 (0.98, 1.12)	1.25*** (1.13, 1.37)
Urban	1.00 (0.8, 1.24)	0.82 (0.60, 1.12)	1.01 (0.88, 1.17)	1.29* (1.05, 1.57)
Rural	0.76 (0.4, 1.43)	0.89 (0.44, 1.77)	0.86 (0.52, 1.4)	1.24 (0.67, 2.28)
Stratification: cancer subtype				
Luminal A	0.79 (0.41, 1.5)	0.69 (0.37, 1.3)	0.84 (0.44, 1.61)	1.17 (0.47, 2.91)
Luminal B	0.86 (0.63, 1.16)	0.90 (0.65, 1.26)	1.04 (0.73, 1.49)	1.64 (0.99, 2.74)
HER2‐Enriched	0.73 (0.19, 2.76)	0.43 (0.1, 1.81)	1.46 (0.29, 7.38)	1.80 (0.37, 8.74)
Triple‐Negative	0.86 (0.47, 1.57)	1.04 (0.52, 2.07)	0.95 (0.53, 1.71)	0.24 (0.03, 1.96)
Stratification: facility type				
Comprehensive Community Cancer Program	0.99 (0.89, 1.11)	0.93 (0.84, 1.04)	1.02 (0.93, 1.11)	1.23** (1.09, 1.40)
Academic/Research Program	0.94 (0.85, 1.05)	0.86* (0.75, 0.99)	1.04 (0.93, 1.16)	1.27** (1.09, 1.47)
Community Cancer Program	0.87* (0.75, 0.99)	0.84 (0.69, 1.03)	1.20* (1.02, 1.41)	1.36* (1.01, 1.82)
Integrated Network Cancer Program	0.97 (0.86, 1.09)	0.77 (0.58, 1.01)	1.04 (0.92, 1.19)	1.19 (0.99, 1.44)

* < 0.05.

** < 0.01.

*** < 0.001.

### 3.3 Stratified Models by Clinical Stage and Molecular Subtype

Compared to patients within 10 miles of the treating facility, patients residing > 20 miles away experienced delays in ToC when diagnosed with early‐stage disease: 8% for Stage 0 (RR: 1.08, 95% CI: 1.05–1.10), 5% for Stage I (RR: 1.05, 95% CI: 1.04–1.06), and 3% for Stage II (RR: 1.03, 95% CI: 1.01–1.05) (Table S4). These corresponded to treatment delays of 3.24 days (95% CI: 2.17–4.30), 1.89 days (95% CI: 1.64–2.14), and 1.32 days (95% CI: 0.53–2.12), respectively. These patterns were not observed in Stage III or IV patients. Delays were also observed among non‐Hispanic Black and Hispanic patients with Stage 0–III disease and among those with Medicaid or no insurance versus private insurance (Table S4).

By molecular subtype, patients with luminal A and B BC residing > 20 miles away experienced treatment delays of 2.81 days (95% CI: 0.31–5.31) and 3.83 days (95% CI: 1.77–5.88), respectively, compared with those ≤ 10 miles (Table 2). No significant delays were observed for HER2‐enriched or triple‐negative subtypes or among patients living 10.1–20.0 miles away. No consistent associations between demographic or clinical factors and ToC were found across subtypes (Table S5).

In stage‐specific Cox models, treatment delays > 12 weeks were associated with higher mortality for Stages 0 (HR: 1.25, 95% CI: 1.06–1.47), I (HR: 1.27, 95% CI: 1.13–1.44), and II (HR: 1.47, 95% CI: 1.26–1.71), but lower mortality for Stage IV (HR: 0.74, 95% CI: 0.55–0.99) (Table 3). Among patients > 20 miles from care, stage I and IV patients had 10% (HR: 0.90, 95% CI: 0.81–0.99) and 18% (HR: 0.82, 95% CI: 0.69–0.99) lower mortality, respectively (Table S6). There were no significant associations between ToC and survival outcomes, nor between GCD and OS across molecular subtypes (Table S7). Differences in predicted survival widened over time for stages I and II but not for Stages 0, III, or IV (Figure S2). There were no significant differences in predicted survival across ToC categories within molecular subtypes (Figure S3).

### 3.4 Stratified Models by Primary Treatment Modality

Among patients residing > 20 miles from their facility, a 5% delay in ToC was observed only for those undergoing surgery (RR: 1.05, 95% CI: 1.04–1.06), corresponding to a 2.2‐day delay (95% CI: 1.83–2.47) (Tables 2 and S8). No significant GCD‐related delays were found for other treatment types. Non‐Hispanic Black patients experienced longer delays across all modalities compared with non‐Hispanic White patients, while higher educational attainment was associated with shorter ToC for surgery, chemotherapy, and radiation (Table S8).

In Cox models stratified by treatment, initiation > 12 weeks was associated with higher mortality among patients receiving surgery (HR: 1.31, 95% CI: 1.19–1.43), compared with initiation within 8 weeks. Even surgery within 8–12 weeks carried elevated risk (HR: 1.09, 95% CI: 1.02–1.16). Patients residing > 20 miles had 11% lower mortality with surgery (HR: 0.89, 95% CI: 0.81–0.98) and 14% lower with chemotherapy (HR: 0.86, 95% CI: 0.75–0.98) compared to those ≤ 10 miles (Table 3). Survival differences widened over time with delayed treatment (Figure S4). Uninsured and Medicaid patients generally had worse OS, and higher Charlson‐Deyo scores and advanced stage were consistently associated with increased mortality (Table S9).

### 3.5 Stratified Models by Geographic and Facility Characteristics

The median time from diagnosis to treatment was shorter for rural patients (31 days) than for those in metropolitan areas (35 days). Across facility types, it ranged from 31 days at CCP to 38 days at ARP. In metropolitan areas, patients residing 10.1–20.0 miles and > 20 miles from their facility experienced treatment delays of 0.46 days (95% CI: 0.14–0.78) and 2.02 days (95% CI: 1.71–2.34), respectively, compared to those ≤ 10 miles away (Table 2). CCCP, serving 43% of patients, was associated with a 1.5‐day delay (95% CI: 0.90–2.14). At ARP, patients residing 10.1–20.0 miles and > 20 miles away experienced additional delays of 0.68 days (95% CI: 0.21–1.41) and 3.13 days (95% CI: 2.45–3.80), respectively. No significant GCD‐related delays were observed for CCP or INCP. Across all areas and facility types, non‐Hispanic Black patients and those with Medicaid experienced longer treatment delays than non‐Hispanic White and privately insured patients Table S10,S11.

Metropolitan patients residing > 20 miles from the treatment facility had a 12% lower mortality risk (HR: 0.88, 95% CI: 0.81–0.96) than those ≤ 10 miles away; no such differences were observed in urban or rural groups (Table 3). Patients treated at ARP and living > 20 miles or treated at CCP and living 10.1–20 miles had lower risks of 14% (HR: 0.86, 95% CI: 0.75–0.99) and 13% (HR: 0.87, 95% CI: 0.75–0.99), respectively (Table S13). Treatment delays > 12 weeks were associated with higher mortality in both metropolitan (HR: 1.25, 95% CI: 1.13–1.37) and urban areas (HR: 1.29, 95% CI: 1.05–1.57) (Table S12). Similar patterns were observed at CCCP, ARP, and CCP, with even delays of 8–12 weeks at CCP linked to 20% higher mortality (HR: 1.20, 95% CI: 1.02–1.41) (Table S13). Survival probability differences widened over time for patients treated later, particularly among those in metropolitan areas (Figure S5) or treated at CCCP or ARP (Figure S6).

## 4 Discussion

Swing up‐to‐date NCDB data, this study evaluated the relationship between travel distance, ToC, and OS in BC, providing nationally representative estimates and laying the groundwork for future mediation analyses. Unlike prior state‐level studies [10, 11, 12, 13, 14, 17, 25, 26, 28, 29, 39, 40], we jointly assessed travel distance, ToC, and OS and quantified marginal treatment delays across subgroups, extending beyond earlier research focused on treatment adherence [5, 6, 7, 12, 13, 16, 25, 26, 27]. We also quantified marginal delays in days across clinical and geographic subgroups, offering a clearer view of how travel burden contributes to disparities in care.

**FIGURE 3:**
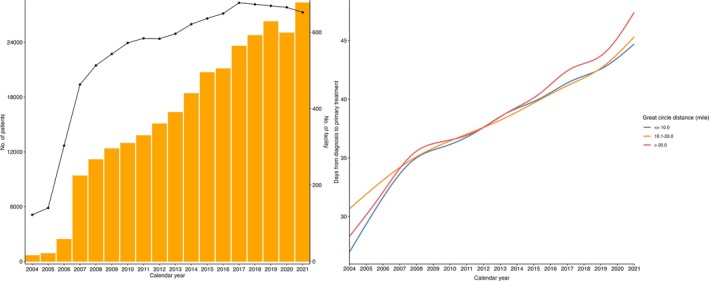
Frequency of patient and facility volumes by calendar year and days from diagnosis to primary treatment, stratified by great circle distance in miles. Plots from left to right show (1) frequency of patients (left y‐axis, orange bars) and clinical facilities (right y‐axis, black line with dots) by calendar year, and (2) days from diagnosis to primary treatment by calendar year, stratified by great circle distance with smooth natural spline curves (blue line for ≤ 10 miles, orange line for 10.1–20 miles, and red line for > 20 miles).

We note that throughout the study period, there was an increasing number of patients and reporting facilities, alongside a gradual rise in time from diagnosis to primary treatment—even when stratified by GCD (Figure 3). In the full cohort, patients residing 10.1–20.0 miles and > 20 miles from their treating facility experienced significant delays in ToC compared to those within 10 miles (Table 2), consistent with previous national [4, 9, 10] and single‐state studies [11, 24]. Additionally, similar to prior studies [19, 20, 21], we identified differences in GCD by living area; while most metropolitan patients traveled ≤ 20 miles, the majority of nonmetropolitan patients—regardless of stage, subtype, or treatment type—traveled > 20 miles (Figure 1).

Notably, only the metropolitan subgroup showed a significant association between GCD and ToC. For clinicians and health planners, this may highlight the importance of addressing even moderate travel distances in metropolitan settings, where logistical barriers may still lead to treatment delays despite greater facility density. Patients from urban and rural areas experienced 5% (RR: 0.95, 95% CI: 0.94–0.96) and 10% (RR: 0.90, 95% CI: 0.87–0.92) shorter time to treatment, respectively, compared with those in metropolitan areas (Table S2), consistent with prior findings [4, 9]. Although GCD was not significantly associated with ToC in non‐metropolitan subgroups, over 80% of these patients traveled > 20 miles (Table S10), reflecting persistent gaps in local care availability [17, 39, 40].

Facility characteristics also influenced ToC. In the full cohort, patients treated at ARP experienced 15% longer time to treatment (RR: 1.15, 95% CI: 1.14–1.16) than those at CCCP (Table S2). This is likely multifactorial and can include longer wait times or scheduling bottlenecks due to higher volumes of patients. Furthermore, significant GCD‐related delays were observed over 10 miles among ARP patients; at CCCP, delays occurred only when GCD exceeded 20 miles (Table S11). For healthcare administrators, improving care coordination and throughput at ARP facilities may help reduce delays—especially for patients traveling greater distances. Expanding service availability at CCCP‐level facilities may also alleviate downstream capacity pressures.

ToC also varied by clinical characteristics. In the full cohort, patients with stage I–IV disease received treatment more quickly than those with stage 0. GCD‐related delays were significant only for early‐stage (Stages 0–II) patients (Table S4), and stratified models showed that these delays were more pronounced among metropolitan than non‐metropolitan patients. This suggests that travel burden may disproportionately affect timely treatment in patients with early‐stage disease, who would benefit most from prompt intervention [28, 29].

Stratified analyses further revealed that patients with luminal A and B subtypes experienced significant delays, while those with more aggressive subtypes did not (Table S5). This may be attributable to a higher proportion of these patients receiving chemotherapy as primary treatment, which was associated with shorter ToC compared to surgery.

Treatment modality was a key determinant of timeliness. Compared with patients undergoing primary surgery, those receiving chemotherapy or endocrine therapy experienced shorter time to treatment, while those receiving radiation experienced longer delays (Table S2). This may reflect the logistical demands of facility‐based treatments. Stratified analyses indicated that travel burden was not significantly associated with timeliness among patients receiving chemotherapy, radiation, or endocrine therapy. However, among those receiving surgery—the primary treatment for 82% of the cohort—residing > 20 miles away was associated with modest but significant delays, consistent with previous studies [4, 6, 9, 10, 25]. This highlights the importance of streamlining access to surgical services for patients with longer travel distances, particularly in high‐volume or resource‐limited systems.

The relationship among OS, travel distance, and delays in ToC varied across patient subgroups and clinical contexts. In the full cohort model, treatment initiation > 12 weeks after diagnosis was significantly associated with worse OS compared to treatment within 10 weeks, consistent with prior studies [3, 41]. Greater travel distance was associated with improved OS, aligning with earlier research [2, 3, 19, 41]. Regarding facility type, no OS advantage was observed for ARP over CCCP.

In stage‐specific models, delayed treatment (> 12 weeks) was associated with worse OS in Stages 0–III (Table S6). However, among patients with stage IV disease, delay was unexpectedly linked to better OS—a finding that differs from prior work [41] and may reflect differences in treatment goals and smaller sample sizes in metastatic settings. Greater GCD was associated with better OS only in stage I and IV patients, suggesting that these subgroups may be driving the overall association.

No significant associations were found between GCD, ToC, and OS by molecular subtype. However, among patients receiving primary surgery or chemotherapy, delays > 12 weeks were linked to worse OS, while no such association was observed in patients treated with radiation or endocrine therapy (Table S9). Higher GCD was also associated with better OS in patients receiving surgery or chemotherapy. Across all OS models, older age, uninsurance or public insurance, higher Charlson‐Deyo scores, and higher tumor grade were consistently associated with worse OS.

This analysis set out to examine the complex interplay between GCD, ToC, geographical setting, facility type, and survival. We identified that GCD > 20 miles was associated with better OS, but only in metropolitan populations. Among patients in metropolitan or urban areas, delays > 12 weeks were associated with worse OS (Table S12). Within facility‐specific models, treatment delays were associated with worse OS in CCCP, ARP, and CCP groups. Notably, greater travel distance was associated with better OS only among ARP patients, supporting prior findings [19] that patients often travel farther to receive high‐quality care at high‐volume centers.

Several limitations should be acknowledged. GCD, while widely used, may not fully capture the complexity of travel burden; alternative metrics such as driving distance or travel time—unavailable in de‐identified NCDB data—may be more accurate [10, 11, 24, 26]. We also observed that patients residing in urban or rural areas were more likely to travel more than 20 miles to receive treatment, suggesting that our categorization may not have fully captured the variance in these smaller subgroups. However, because the majority of participants in our cohort lived in metropolitan areas (85%) and more than half (53%) resided within 10 miles of their treating facility, the chosen cutoffs reflect the predominant distribution of travel burden in this population. Future research focusing specifically on rural and urban populations will be important to determine whether alternative distance thresholds provide a more accurate representation of travel burden in these settings. Observed travel distance may also reflect patient choice rather than true burden, as some patients with greater resources may travel farther to access specialized care [42, 43, 44]; however, the NCDB does not capture whether treatment occurred at the nearest facility. Treatment subtypes were not distinguished (e.g., contralateral prophylactic mastectomy or neoadjuvant chemotherapy) [5, 7, 12, 25, 26], as our intent was to offer a high‐level national perspective rather than granular clinical detail. Finally, NCDB's observational design limits causal inference and control for unmeasured confounding.

## 5 Conclusion

Because of the complex interplay of factors affecting travel burden, a one‐size‐fits‐all approach is insufficient. Multiple systematic reviews have shown that existing interventions targeting travel burden remain inadequate [19, 20, 21, 22, 23]. The impact of travel burden and timeliness of care varies by geography, facility type, and clinical context. Interventions must be tailored to local capacity, patient demographics, and disease profiles to promote equitable, timely access to breast cancer care. Policymakers should recognize that delays disproportionately affect early‐stage patients, especially those in metropolitan areas. Future studies are needed to better evaluate causal pathways linking travel burden, care delays, and survival.

## Author Contributions

**Yongzhe Wang:** conceptualization (equal), data curation (equal), formal analysis (equal), investigation (equal), methodology (equal), project administration (equal), software (equal), validation (equal), visualization (equal), writing – original draft (equal), writing – review and editing (equal). **Christine M. Quinones:** data curation (equal), investigation (equal), project administration (equal), validation (equal), writing – review and editing (equal). **Elizabeth Gonzalez:** investigation (equal), validation (equal), writing – original draft (equal), writing – review and editing (equal). **Preeti Farmah:** investigation (equal), validation (equal), writing – review and editing (equal). **Hans F. Schoellhammer:** investigation (equal), validation (equal), writing – review and editing (equal). **Lorena Gonzalez:** investigation (equal), validation (equal), writing – review and editing (equal). **Nikita Shah:** investigation (equal), validation (equal), writing – review and editing (equal). **Katharine Schulz‐Costello:** investigation (equal), validation (equal), writing – review and editing (equal). **Jennifer Tseng:** investigation (equal), validation (equal), writing – review and editing (equal). **Veronica C. Jones:** conceptualization (equal), data curation (equal), funding acquisition (equal), investigation (equal), methodology (equal), project administration (equal), resources (equal), supervision (equal), validation (equal), writing – original draft (equal), writing – review and editing (equal).

## Ethics Statement

Given that the dataset consists of deidentified patient information, the study was deemed exempt from institutional review board oversight.

## Consent

Precis: The relationship between travel burden, timeliness of care, and overall survival in breast cancer varies by geography, facility type, and clinical context. Patients from metropolitan areas, with early‐stage disease, undergoing surgery, or treated at high‐volume facilities face greater delays, and delays beyond 12 weeks are generally associated with worse survival outcomes.

## Conflicts of Interest

The authors declare no conflicts of interest.

## Supporting information


**Table S1:** Detailed information on variables used in the study from the National Cancer Database.
**Table S2:** Estimated rate ratios with 95% confidence intervals from adjusted Poisson regression models via generalized estimating equations for overall population.
**Table S3:** Estimated hazard ratios with 95% confidence intervals from adjusted Cox models for overall population.
**Table S4:** Estimated rate ratios with 95% confidence intervals from adjusted Poisson regression models via generalized estimating equations stratified by cancer stage.
**Table S5:** Estimated rate ratios with 95% confidence intervals from adjusted Poisson regression models via generalized estimating equations stratified by molecular subtype.
**Table S6:** Estimated hazard ratios with 95% confidence intervals from adjusted Cox models stratified by cancer stage.
**Table S7:** Estimated hazard ratios with 95% confidence intervals from adjusted Cox models stratified by molecular subtype.
**Table S8:** Estimated rate ratios with 95% confidence intervals from adjusted Poisson regression models via generalized estimating equations stratified by primary treatment.
**Table S9:** Estimated hazard ratios with 95% confidence intervals from adjusted Cox models stratified by primary treatment.
**Table S10:** Estimated rate ratios with 95% confidence intervals from adjusted Poisson regression models via generalized estimating equations stratified by living area.
**Table S11:** Estimated rate ratios with 95% confidence intervals from adjusted Poisson regression models via generalized estimating equations stratified by facility type.
**Table S12:** Estimated hazard ratios with 95% confidence intervals from adjusted Cox models stratified by living area.
**Table S13:** Estimated hazard ratios with 95% confidence intervals from adjusted Cox models stratified by facility type.
**Figure S1:** Predicted survival probability differences by timeliness of care categories from the adjusted Cox model for the overall population.
**Figure S2:** Predicted survival probability differences by timeliness of care categories from adjusted Cox models for the cancer stage (Stage 0, I, II, III, IV).
**Figure S3:** Predicted survival probability differences by timeliness of care categories from adjusted Cox models for the molecular subtype (luminal A, luminal B, HER2‐Enriched, triple‐negative).
**Figure S4:** Predicted survival probability differences by timeliness of care categories from adjusted Cox models for the primary modality (surgery, chemotherapy, radiation, endocrine therapy).
**Figure S5:** Predicted survival probability differences by timeliness of care categories from adjusted Cox models for the living area (metro, urban, rural).
**Figure S6:** Predicted survival probability differences by timeliness of care categories from adjusted Cox models for the facility type (comprehensive community cancer program, academic/research program, community cancer program, integrated network cancer program).

## Data Availability

The National Cancer Database is publicly accessible through an application on the website: https://www.facs.org/quality‐programs/cancer‐programs/national‐cancer‐database/.
