# Supplementary material for: Impact of Travel Burden on Timeliness of Care and Overall Survival for Breast Cancer: A National Cancer Database Analysis
Source: Cancer Med. 2025 Nov 10;14(21):e71354. doi: 10.1002/cam4.71354 (PMC12599556; doi:10.1002/cam4.71354)
Supplement: Supplementary file 1 — Table S1: Detailed information on variables used in the study from the National Cancer Database. Table S2: Estimated rate ratios with 95% confidence intervals from adjusted Poisson regression models via generalized estimating equations for overall population. Table S3: Estimated hazard ratios with 95% confidence intervals from adjusted Cox models for overall population. Table S4: Estimated rate ratios with 95% confidence intervals from adjusted Poisson regression models via generalized estimating equations stratified by cancer stage. Table S5: Estimated rate ratios with 95% confidence intervals from adjusted Poisson regression models via generalized estimating equations stratified by molecular subtype. Table S6: Estimated hazard ratios with 95% confidence intervals from adjusted Cox models stratified by cancer stage. Table S7: Estimated hazard ratios with 95% confidence intervals from adjusted Cox models stratified by molecular subtype. Table S8: Estimated rate ratios with 95% confidence intervals from adjusted Poisson regression models via generalized estimating equations stratified by primary treatment. Table S9: Estimated hazard ratios with 95% confidence intervals from adjusted Cox models stratified by primary treatment. Table S10: Estimated rate ratios with 95% confidence intervals from adjusted Poisson regression models via generalized estimating equations stratified by living area. Table S11: Estimated rate ratios with 95% confidence intervals from adjusted Poisson regression models via generalized estimating equations stratified by facility type. Table S12: Estimated hazard ratios with 95% confidence intervals from adjusted Cox models stratified by living area. Table S13: Estimated hazard ratios with 95% confidence intervals from adjusted Cox models stratified by facility type. Figure S1: Predicted survival probability differences by timeliness of care categories from the adjusted Cox model for the overall population. Figure S2: Predicted survival probabilit [file CAM4-14-e71354-s001.docx]

**Impact of travel burden on timeliness of care and overall survival for breast cancer: a national cancer database analysis**

**Supplementary Materials**

**Content**

**Supplementary table 1** - Detailed information on variables used in the study from the National Cancer Database

**Supplementary table 2** - Estimated rate ratios with 95% confidence intervals from adjusted Poisson regression models via generalized estimating equations for overall population

**Supplementary table 3** - Estimated hazard ratios with 95% confidence intervals from adjusted Cox models for overall population

**Supplementary table 4** - Estimated rate ratios with 95% confidence intervals from adjusted Poisson regression models via generalized estimating equations stratified by cancer stage

**Supplementary table 5** - Estimated rate ratios with 95% confidence intervals from adjusted Poisson regression models via generalized estimating equations stratified by molecular subtype

**Supplementary table 6** - Estimated hazard ratios with 95% confidence intervals from adjusted Cox models stratified by cancer stage

**Supplementary table 7** - Estimated hazard ratios with 95% confidence intervals from adjusted Cox models stratified by molecular subtype

**Supplementary table 8** - Estimated rate ratios with 95% confidence intervals from adjusted Poisson regression models via generalized estimating equations stratified by primary treatment

**Supplementary table 9** - Estimated hazard ratios with 95% confidence intervals from adjusted Cox models stratified by primary treatment

**Supplementary table 10** - Estimated rate ratios with 95% confidence intervals from adjusted Poisson regression models via generalized estimating equations stratified by living area

**Supplementary table 11** - Estimated rate ratios with 95% confidence intervals from adjusted Poisson regression models via generalized estimating equations stratified by facility type

**Supplementary table 12** - Estimated hazard ratios with 95% confidence intervals from adjusted Cox models stratified by living area

**Supplementary table 13** - Estimated hazard ratios with 95% confidence intervals from adjusted Cox models stratified by facility type

**Supplementary figure 1** - Predicted survival probability differences by timeliness of care categories from adjusted Cox models for the overall population

**Supplementary figure 2** - Predicted survival probability differences by timeliness of care categories from adjusted Cox models for the cancer stage

**Supplementary figure 3** - Predicted survival probability differences by timeliness of care categories from adjusted Cox models for the molecular subtype

**Supplementary figure 4** - Predicted survival probability differences by timeliness of care categories from adjusted Cox models for the primary modality

**Supplementary figure 5** - Predicted survival probability differences by timeliness of care categories from adjusted Cox models for the living area

**Supplementary figure 6** - Predicted survival probability differences by timeliness of care categories from adjusted Cox models for the facility type

**Table S1**: Detailed information on variables used in the study from the National Cancer Database

| **Variable** | **Detail** | **Variable code in NCDB**^a^ |
| --- | --- | --- |
| Great circle distance in miles | <=10 miles 10.1-20 miles >20 miles | CROWFLY |
| Age at diagnosis | 40-49 50-59 60-69 70-79 80+ | AGE |
| Education: percent no high school degree | No HSD >=15.3% No HSD 9.1% - 15.2% No HSD 5.0% - 9.0% No HSD < 5.0% Unknown | NO_HSD_QUAR_2020 |
| Median household income | < $46,227 $46,227 - $57,856 $57,857 - $74,062 >= $74,063 Unknown | MED_INC_QUAR_2020 |
| Insurance | Private Insurance No Medicaid Medicare Other Government Unknown | INSURANCE_STATUS |
| Race and ethnicity | White, NH Black, NH Hispanic Asian, NH AIAN, NH NHPI, NH Other Unknown | RACE SPANISH_HISPANIC_ORIGIN |
| Living area | Metro Urban Rural Unknown | UR_CD_13 |
| Facility type | Comprehensive Community Cancer Program Academic/Research Program Community Cancer Program Integrated Network Cancer Program | FACILITY_TYPE_CD |
| Facility location | West (Mountain, Pacific) South (South Atlantic, East South Central, West South Central) Midwest (East North Central, West North Central) Northeast (New England, Middle Atlantic) | FACILITY_LOCATION_CD |
| Charlson-Deyo index | 0 1 2 3 | CDCC_TOTAL_BEST |
| Tumor grade | Grade I Grade II Grade III Grade IV Unknown | GRADE GRADE_CLIN |
| Cancer stage | Stage 0 Stage I Stage II Stage III Stage IV Unknown | ANALYTIC_STAGE_GROUP |
| Primary treatment | Surgery Chemotherapy Radiation Endocrine therapy | DX_DEFSURG_STARTED_DAYS DX_CHEMO_STARTED_DAYS DX_RAD_STARTED_DAYS DX_HORMONE_STARTED_DAYS |
| Cancer subtype | Luminal A Luminal B HER2-Enriched Triple-Negative Unknown | ER_PERCENT_POS_OR_RNG PR_PERCENT_POS_OR_RNG HER2_ISH_DUAL_NUM KI67 |
| Overall survival status | Alive Censored Dead | PUF_VITAL_STATUS |

^a^ Additional details regarding variable definitions are available in the Participant User File (PUF) data dictionary provided by the National Cancer Database: https://www.facs.org/quality-programs/cancer-programs/national-cancer-database/puf/

**Table S2**: Estimated rate ratios with 95% confidence intervals from adjusted Poisson regression models via generalized estimating equations for overall population

| **Characteristics** | **Rate ratio with 95% confidence interval (n = 283,166)** |
| --- | --- |
| **Primary treatment** | |
| Surgery | 1 |
| Chemotherapy | 0.91*** (0.90, 0.92) |
| Radiation | 1.27*** (1.23, 1.33) |
| Endocrine therapy | 0.90*** (0.88, 0.92) |
| **Great circle distance in miles** | |
| < 10.0 | 1 |
| 10.1-20.0 | 1.01* (1.00, 1.02) |
| > 20.0 | 1.05*** (1.04, 1.06) |
| **Race and ethnicity** | |
| White, Non-Hispanic | 1 |
| Black, Non-Hispanic | 1.18*** (1.17, 1.20) |
| Hispanic | 1.14*** (1.10, 1.18) |
| Asian, Non-Hispanic | 1.04*** (1.02, 1.05) |
| AIAN, Non-Hispanic | 1.13*** (1.06, 1.21) |
| NHPI, Non-Hispanic | 1.03 (0.97, 1.09) |
| Other | 1.11*** (1.07, 1.15) |
| **Age at diagnosis** | |
| < 50 | 1 |
| 50-59 | 0.95*** (0.94, 0.96) |
| 60-69 | 0.92*** (0.91, 0.93) |
| 70-79 | 0.88*** (0.87, 0.89) |
| 80+ | 0.86*** (0.85, 0.88) |
| **Education: percent no high school degree** | |
| No HSD >=15.3% | 1 |
| No HSD 9.1% - 15.2% | 0.97*** (0.96, 0.98) |
| No HSD 5.0% - 9.0% | 0.96*** (0.94, 0.97) |
| No HSD < 5.0% | 0.94*** (0.93, 0.95) |
| **Median household income** | |
| < $46,227 | 1 |
| $46,227 - $57,856 | 1.01* (1.00, 1.02) |
| $57,857 - $74,062 | 1.00 (0.99, 1.02) |
| >= $74,063 | 1.01 (0.99, 1.02) |
| **Insurance status** | |
| Private | 1 |
| Uninsured | 1.19*** (1.16, 1.22) |
| Medicaid | 1.15*** (1.13, 1.17) |
| Medicare | 1.03*** (1.02, 1.04) |
| Other Government | 1.08*** (1.04, 1.11) |
| **Living area** | |
| Metro | 1 |
| Urban | 0.95*** (0.94, 0.96) |
| Rural | 0.90*** (0.87, 0.92) |
| **Facility type** | |
| Comprehensive Community Cancer Program | 1 |
| Academic/Research Program | 1.15*** (1.14, 1.16) |
| Community Cancer Program | 0.99* (0.97, 1.00) |
| Integrated Network Cancer Program | 1.07*** (1.06, 1.08) |
| **Facility location** | |
| West | 1 |
| South | 0.97*** (0.96, 0.98) |
| Midwest | 0.89*** (0.88, 0.90) |
| Northeast | 1.02*** (1.01, 1.03) |
| **Charlson-Deyo score** | |
| 0 | 1 |
| 1 | 1.03*** (1.02, 1.04) |
| 2 | 1.04*** (1.03, 1.06) |
| 3 | 1.10*** (1.07, 1.13) |
| **Cancer stage** | |
| 0 | 1 |
| I | 0.93*** (0.92, 0.94) |
| II | 0.96*** (0.95, 0.97) |
| III | 0.97*** (0.95, 0.98) |
| IV | 0.81*** (0.78, 0.84) |
| **Cancer subtype** | |
| Luminal A | 1 |
| Luminal B | 0.99 (0.96, 1.01) |
| HER2-Enriched | 1.01 (0.95, 1.07) |
| Triple-Negative | 0.92*** (0.89, 0.96) |
| **Tumor grade** | |
| I | 1 |
| II | 1.03*** (1.02, 1.04) |
| III | 0.99 (0.98, 1.00) |
| IV | 1.06* (1.01, 1.12) |
| **Year of diagnosis** | 1.02*** (1.02, 1.02) |

Significance: * < 0.05, ** < 0.01, *** < 0.001

**Table S3**: Estimated hazard ratios with 95% confidence intervals from adjusted Cox models for overall population

| **Characteristics** | **Hazard ratio with 95% confidence interval (n = 283,166)** |
| --- | --- |
| **Primary treatment** | |
| Surgery | 1 |
| Chemotherapy | 1.41*** (1.31, 1.5) |
| Radiation | 1.44*** (1.24, 1.68) |
| Endocrine therapy | 1.36*** (1.24, 1.49) |
| **Great circle distance in miles** | |
| < 10.0 | 1 |
| 10.1 - 20.0 | 0.95 (0.9, 1.01) |
| > 20.0 | 0.88** (0.81, 0.96) |
| **Weeks from diagnosis to initial treatment** | |
| < 8 | 1 |
| 8 - 12 | 1.04 (0.98, 1.1) |
| > 12 | 1.24*** (1.14, 1.35) |
| **Race and ethnicity** | |
| White, Non-Hispanic | 1 |
| Black, Non-Hispanic | 0.96 (0.88, 1.06) |
| Hispanic | 0.76** (0.62, 0.94) |
| Asian, Non-Hispanic | 0.62*** (0.53, 0.73) |
| AIAN, Non-Hispanic | 1.26 (0.94, 1.68) |
| NHPI, Non-Hispanic | 1.45** (1.1, 1.89) |
| Other | 0.75 (0.55, 1.01) |
| **Age at diagnosis** | |
| < 50 | 1 |
| 50-59 | 1.39*** (1.29, 1.5) |
| 60-69 | 2.06*** (1.9, 2.23) |
| 70-79 | 4.12*** (3.72, 4.58) |
| 80+ | 11.42*** (10.17, 12.83) |
| **Education: percent no high school degree** | |
| No HSD >=15.3% | 1 |
| No HSD 9.1% - 15.2% | 1.03 (0.93, 1.14) |
| No HSD 5.0% - 9.0% | 0.94 (0.84, 1.07) |
| No HSD < 5.0% | 0.86* (0.76, 0.98) |
| **Median household income** | |
| < $46,227 | 1 |
| $46,227 - $57,856 | 0.91 (0.81, 1.03) |
| $57,857 - $74,062 | 0.84** (0.74, 0.94) |
| >= $74,063 | 0.77*** (0.67, 0.89) |
| **Insurance status** | |
| Private | 1 |
| Uninsured | 1.58*** (1.35, 1.84) |
| Medicaid | 1.69*** (1.54, 1.85) |
| Medicare | 1.43*** (1.33, 1.53) |
| Other Government | 1.23* (1.01, 1.49) |
| **Living area** | |
| Metro | 1 |
| Urban | 1.05 (0.93, 1.18) |
| Rural | 1.10 (0.92, 1.32) |
| **Facility type** | |
| Comprehensive Community Cancer Program | 1 |
| Academic/Research Program | 0.91 (0.75, 1.11) |
| Community Cancer Program | 1.33** (1.11, 1.59) |
| Integrated Network Cancer Program | 1.16 (0.93, 1.45) |
| **Facility location** | |
| West | 1 |
| South | 0.95 (0.73, 1.24) |
| Midwest | 0.92 (0.7, 1.2) |
| Northeast | 1.08 (0.79, 1.48) |
| **Charlson-Deyo score** | |
| 0 | 1 |
| 1 | 1.48*** (1.4, 1.57) |
| 2 | 1.99*** (1.81, 2.19) |
| 3 | 2.83*** (2.49, 3.22) |
| **Cancer stage** | |
| 0 | 1 |
| I | 1.26*** (1.19, 1.34) |
| II | 1.79*** (1.68, 1.9) |
| III | 3.13*** (2.88, 3.39) |
| IV | 10.07*** (9.02, 11.24) |
| **Tumor grade** | |
| I | 1 |
| II | 1.12*** (1.07, 1.17) |
| III | 1.28*** (1.21, 1.35) |
| IV | 1.30* (1.02, 1.66) |
| **Year of diagnosis** | 1.34*** (1.22, 1.47) |

Significance: * < 0.05, ** < 0.01, *** < 0.001

**Table S4**: Estimated rate ratios with 95% confidence intervals from adjusted Poisson regression models via generalized estimating equations stratified by cancer stage

| **Characteristics** | **Rate ratio with 95% confidence interval** | | | | |
| --- | --- | --- | --- | --- | --- |
|  | **Stage 0 (n = 56,235)** | **Stage I (n = 142,530)** | **Stage II (n = 54,119)** | **Stage III (n = 16,158)** | **Stage IV (n = 5,945)** |
| **Primary treatment** | | | | | |
| Surgery | 1 | 1 | 1 | 1 | 1 |
| Chemotherapy | 0.74*** (0.71, 0.77) | 0.93*** (0.92, 0.95) | 0.89*** (0.88, 0.91) | 0.80*** (0.78, 0.83) | 0.90 (0.80, 1.02) |
| Radiation | 1.21*** (1.10, 1.32) | 1.13*** (1.10, 1.17) | 1.33*** (1.12, 1.57) | 1.86** (1.24, 2.80) | 1.12 (0.63, 2.01) |
| Endocrine therapy | 0.98 (0.91, 1.06) | 0.90*** (0.87, 0.93) | 0.89*** (0.83, 0.95) | 0.73*** (0.68, 0.79) | 0.83* (0.71, 0.97) |
| **Great circle distance in miles** | | | | | |
| < 10.0 | 1 | 1 | 1 | 1 | 1 |
| 10.1-20.0 | 1.01 (0.99, 1.04) | 1.01* (1.00, 1.02) | 1.00 (0.98, 1.02) | 1.02 (0.98, 1.05) | 0.89 (0.76, 1.04) |
| > 20.0 | 1.08*** (1.05, 1.10) | 1.05*** (1.04, 1.06) | 1.03** (1.01, 1.05) | 1.02 (0.99, 1.06) | 0.95 (0.84, 1.09) |
| **Race and ethnicity** | | | | | |
| White, Non-Hispanic | 1 | 1 | 1 | 1 | 1 |
| Black, Non-Hispanic | 1.20*** (1.17, 1.23) | 1.17*** (1.16, 1.19) | 1.17*** (1.14, 1.20) | 1.19*** (1.13, 1.24) | 1.20* (1.01, 1.42) |
| Hispanic | 1.14* (1.02, 1.28) | 1.15*** (1.11, 1.19) | 1.10*** (1.04, 1.16) | 1.19*** (1.08, 1.31) | 1.50 (0.87, 2.59) |
| Asian, Non-Hispanic | 1.02 (0.99, 1.06) | 1.04*** (1.02, 1.07) | 1.04 (0.99, 1.09) | 0.99 (0.91, 1.08) | 1.07 (0.83, 1.39) |
| AIAN, Non-Hispanic | 1.13 (0.95, 1.35) | 1.14** (1.05, 1.24) | 1.10 (0.95, 1.27) | 1.29** (1.07, 1.57) | 2.09*** (1.37, 3.18) |
| NHPI, Non-Hispanic | 1.07 (0.90, 1.27) | 1.07 (0.98, 1.16) | 1.03 (0.91, 1.15) | 0.94 (0.74, 1.18) | 0.61 (0.34, 1.13) |
| Other | 1.13** (1.05, 1.22) | 1.12*** (1.06, 1.18) | 1.08 (0.99, 1.18) | 1.17* (1.02, 1.35) | 1.23 (0.76, 1.98) |
| **Age at diagnosis** | | | | | |
| < 50 | 1 | 1 | 1 | 1 | 1 |
| 50-59 | 0.91*** (0.89, 0.94) | 0.93*** (0.92, 0.95) | 0.99 (0.97, 1.00) | 1.03 (0.99, 1.06) | 0.97 (0.82, 1.14) |
| 60-69 | 0.87*** (0.85, 0.90) | 0.90*** (0.89, 0.91) | 0.96*** (0.94, 0.98) | 1.02 (0.98, 1.06) | 0.95 (0.78, 1.14) |
| 70-79 | 0.84*** (0.81, 0.87) | 0.87*** (0.86, 0.89) | 0.91*** (0.88, 0.94) | 1.00 (0.94, 1.05) | 0.85 (0.66, 1.11) |
| 80+ | 0.80*** (0.76, 0.84) | 0.86*** (0.84, 0.88) | 0.88*** (0.85, 0.92) | 0.89** (0.83, 0.96) | 0.86 (0.58, 1.29) |
| **Education: percent no high school degree** | | | | | |
| No HSD >=15.3% | 1 | 1 | 1 | 1 | 1 |
| No HSD 9.1% - 15.2% | 0.99 (0.96, 1.02) | 0.97*** (0.96, 0.99) | 0.96*** (0.94, 0.98) | 0.95* (0.91, 1.00) | 0.81* (0.69, 0.96) |
| No HSD 5.0% - 9.0% | 0.98 (0.95, 1.01) | 0.95*** (0.94, 0.97) | 0.95*** (0.93, 0.98) | 0.93** (0.88, 0.98) | 0.81* (0.67, 0.98) |
| No HSD < 5.0% | 0.99 (0.96, 1.03) | 0.94*** (0.92, 0.96) | 0.92*** (0.89, 0.94) | 0.90*** (0.85, 0.95) | 0.68*** (0.55, 0.84) |
| **Median household income** | | | | | |
| < $46,227 | 1 | 1 | 1 | 1 | 1 |
| $46,227 - $57,856 | 1.01 (0.98, 1.04) | 1.00 (0.99, 1.02) | 1.04*** (1.02, 1.07) | 1.02 (0.97, 1.07) | 1.08 (0.89, 1.32) |
| $57,857 - $74,062 | 1.01 (0.98, 1.04) | 0.99 (0.97, 1.01) | 1.02 (0.99, 1.04) | 1.02 (0.96, 1.08) | 1.12 (0.94, 1.35) |
| >= $74,063 | 1.02 (0.99, 1.06) | 0.99 (0.97, 1.01) | 1.04* (1.01, 1.07) | 1.01 (0.95, 1.07) | 1.13 (0.91, 1.41) |
| **Insurance status** | | | | | |
| Private | 1 | 1 | 1 | 1 | 1 |
| Uninsured | 1.16*** (1.08, 1.25) | 1.22*** (1.18, 1.27) | 1.21*** (1.14, 1.27) | 1.21*** (1.11, 1.32) | 1.08 (0.84, 1.39) |
| Medicaid | 1.13*** (1.08, 1.17) | 1.15*** (1.12, 1.17) | 1.20*** (1.16, 1.24) | 1.16*** (1.11, 1.22) | 1.09 (0.91, 1.30) |
| Medicare | 1.01 (0.99, 1.04) | 1.02** (1.01, 1.03) | 1.05** (1.02, 1.08) | 1.04 (1.00, 1.09) | 1.08 (0.89, 1.32) |
| Other Government | 1.03 (0.95, 1.10) | 1.09*** (1.05, 1.13) | 1.10** (1.04, 1.18) | 1.14* (1.02, 1.27) | 0.54* (0.33, 0.87) |
| **Living area** | | | | | |
| Metro | 1 | 1 | 1 | 1 | 1 |
| Urban | 0.93*** (0.91, 0.96) | 0.96*** (0.94, 0.97) | 0.95*** (0.93, 0.98) | 0.96 (0.91, 1.00) | 1.00 (0.84, 1.20) |
| Rural | 0.90** (0.84, 0.96) | 0.90*** (0.87, 0.93) | 0.87*** (0.83, 0.91) | 0.95 (0.86, 1.05) | 0.92 (0.72, 1.17) |
| **Facility type** | | | | | |
| Comprehensive Community Cancer Program | 1 | 1 | 1 | 1 | 1 |
| Academic/Research Program | 1.17*** (1.15, 1.20) | 1.15*** (1.14, 1.16) | 1.16*** (1.14, 1.18) | 1.16*** (1.12, 1.20) | 1.16* (1.02, 1.32) |
| Community Cancer Program | 0.97 (0.94, 1.01) | 0.97*** (0.96, 0.99) | 1.02 (0.99, 1.05) | 1.03 (0.98, 1.09) | 1.01 (0.79, 1.27) |
| Integrated Network Cancer Program | 1.08*** (1.05, 1.11) | 1.06*** (1.05, 1.07) | 1.08*** (1.04, 1.11) | 1.09*** (1.05, 1.13) | 1.16 (0.99, 1.36) |
| **Facility location** | | | | | |
| West | 1 | 1 | 1 | 1 | 1 |
| South | 0.94*** (0.92, 0.97) | 0.98** (0.97, 0.99) | 0.97* (0.94, 0.99) | 0.97 (0.93, 1.02) | 0.95 (0.80, 1.12) |
| Midwest | 0.87*** (0.84, 0.89) | 0.91*** (0.90, 0.92) | 0.89*** (0.87, 0.91) | 0.87*** (0.83, 0.91) | 0.95 (0.79, 1.13) |
| Northeast | 0.96** (0.93, 0.99) | 1.04*** (1.03, 1.06) | 1.05*** (1.02, 1.08) | 1.05 (0.99, 1.10) | 0.99 (0.79, 1.22) |
| **Charlson-Deyo score** | | | | | |
| 0 | 1 | 1 | 1 | 1 | 1 |
| 1 | 1.02 (0.99, 1.04) | 1.03*** (1.02, 1.05) | 1.03** (1.01, 1.05) | 1.02 (0.98, 1.07) | 0.96 (0.83, 1.10) |
| 2 | 1.01 (0.96, 1.06) | 1.06*** (1.04, 1.09) | 1.06** (1.02, 1.10) | 0.99 (0.93, 1.06) | 1.10 (0.85, 1.44) |
| 3 | 1.12** (1.04, 1.22) | 1.11*** (1.08, 1.15) | 1.06 (0.99, 1.12) | 1.08 (0.98, 1.19) | 1.15 (0.72, 1.84) |
| **Tumor grade** | | | | | |
| I | 1 | 1 | 1 | 1 | 1 |
| II | 1.14*** (1.10, 1.18) | 1.02*** (1.01, 1.03) | 0.99 (0.97, 1.01) | 1.00 (0.96, 1.04) | 0.79* (0.65, 0.95) |
| III | 1.18*** (1.14, 1.22) | 0.97*** (0.96, 0.99) | 0.91*** (0.89, 0.93) | 0.93** (0.89, 0.97) | 0.70*** (0.58, 0.85) |
| IV | 1.17*** (1.09, 1.26) | 1.02 (0.90, 1.17) | 1.03 (0.85, 1.24) | 0.83 (0.63, 1.10) | 0.72 (0.45, 1.15) |
| **Year of diagnosis** | 1.02*** (1.02, 1.03) | 1.02*** (1.02, 1.02) | 1.02*** (1.02, 1.02) | 1.03*** (1.02, 1.03) | 1.02** (1.00, 1.03) |

Significance: * < 0.05, ** < 0.01, *** < 0.001

**Table S5**: Estimated rate ratios with 95% confidence intervals from adjusted Poisson regression models via generalized estimating equations stratified by molecular subtype

| **Characteristics** | **Rate ratio with 95% confidence interval** | | | |
| --- | --- | --- | --- | --- |
|  | **Luminal A (n = 3,943)** | **Luminal B (n = 7,015)** | **HER2-Enriched (n = 785)** | **Triple-Negative (n = 1,721)** |
| **Primary treatment** | | | | |
| Surgery | 1 | 1 | 1 | 1 |
| Chemotherapy | 0.94 (0.82, 1.08) | 0.87*** (0.83, 0.91) | 0.78** (0.67, 0.91) | 0.83*** (0.76, 0.90) |
| Radiation | 1.00 (0.80, 1.24) | 1.18 (0.77, 1.81) | 3.46*** (1.90, 6.30) | 1.44 (0.98, 2.11) |
| Endocrine therapy | 0.79*** (0.71, 0.88) | 0.72*** (0.66, 0.79) | - | 0.18*** (0.12, 0.27) |
| **Great circle distance in miles** | | | | |
| < 10.0 | 1 | 1 | 1 | 1 |
| 10.1-20.0 | 1.04 (0.99, 1.10) | 1.03 (0.98, 1.09) | 1.04 (0.93, 1.16) | 1.01 (0.95, 1.09) |
| > 20.0 | 1.06* (1.01, 1.12) | 1.09*** (1.04, 1.14) | 1.13 (0.95, 1.34) | 1.06 (0.97, 1.15) |
| **Race and ethnicity** | | | | |
| White, Non-Hispanic | 1 | 1 | 1 | 1 |
| Black, Non-Hispanic | 1.17*** (1.08, 1.27) | 1.09** (1.04, 1.16) | 1.10 (0.96, 1.26) | 1.12** (1.03, 1.21) |
| Hispanic | 1.05 (0.89, 1.23) | 1.22** (1.07, 1.38) | 1.33 (0.87, 2.03) | 1.07 (0.91, 1.24) |
| Asian, Non-Hispanic | 1.01 (0.93, 1.10) | 1.09 (0.97, 1.22) | 0.73 (0.52, 1.02) | 1.04 (0.92, 1.18) |
| AIAN, Non-Hispanic | 0.82 (0.48, 1.41) | 0.98 (0.82, 1.17) | 0.53*** (0.42, 0.66) | 0.88 (0.70, 1.11) |
| NHPI, Non-Hispanic | 1.40 (0.96, 2.02) | 1.52* (1.06, 2.17) | 0.68** (0.52, 0.88) | 0.93 (0.69, 1.26) |
| Other | 1.02 (0.84, 1.25) | 0.92 (0.82, 1.03) | 1.82* (1.08, 3.07) | 1.10 (0.87, 1.38) |
| **Education: percent no high school degree** | | | | |
| No HSD >=15.3% | 1 | 1 | 1 | 1 |
| No HSD 9.1% - 15.2% | 0.96 (0.89, 1.05) | 0.95 (0.89, 1.02) | 0.89 (0.76, 1.04) | 1.01 (0.93, 1.10) |
| No HSD 5.0% - 9.0% | 0.95 (0.87, 1.03) | 0.95 (0.88, 1.01) | 0.86 (0.71, 1.05) | 1.00 (0.91, 1.11) |
| No HSD < 5.0% | 0.92 (0.84, 1.01) | 0.94 (0.87, 1.01) | 0.87 (0.70, 1.09) | 1.03 (0.92, 1.14) |
| **Median household income** | | | | |
| < $46,227 | 1 | 1 | 1 | 1 |
| $46,227 - $57,856 | 0.99 (0.90, 1.09) | 0.99 (0.91, 1.07) | 1.21* (1.00, 1.47) | 1.02 (0.93, 1.11) |
| $57,857 - $74,062 | 0.93 (0.84, 1.02) | 0.93* (0.87, 0.99) | 1.10 (0.92, 1.32) | 0.99 (0.90, 1.10) |
| >= $74,063 | 0.98 (0.88, 1.09) | 0.95 (0.89, 1.02) | 1.16 (0.92, 1.46) | 0.91 (0.82, 1.01) |
| **Insurance status** | | | | |
| Private | 1 | 1 | 1 | 1 |
| Uninsured | 1.22 (0.95, 1.57) | 1.21** (1.06, 1.37) | 1.31* (1.04, 1.67) | 1.13 (0.93, 1.37) |
| Medicaid | 1.13* (1.02, 1.24) | 1.13** (1.05, 1.22) | 1.18 (0.94, 1.49) | 1.40*** (1.24, 1.58) |
| Medicare | 0.98 (0.92, 1.05) | 1.05 (1.00, 1.11) | 1.06 (0.91, 1.23) | 1.04 (0.95, 1.14) |
| Other Government | 1.29 (0.92, 1.81) | 1.11 (0.97, 1.25) | 0.85 (0.62, 1.16) | 1.10 (0.77, 1.56) |
| **Living area** | | | | |
| Metro | 1 | 1 | 1 | 1 |
| Urban | 0.93 (0.86, 1.01) | 0.94* (0.88, 1.00) | 0.79* (0.65, 0.97) | 0.93 (0.83, 1.03) |
| Rural | 0.87* (0.76, 0.99) | 0.87 (0.76, 1.00) | 0.90 (0.66, 1.23) | 0.80 (0.58, 1.11) |
| **Facility type** | | | | |
| Comprehensive Community Cancer Program | 1 | 1 | 1 | 1 |
| Academic/Research Program | 1.13*** (1.06, 1.20) | 1.10*** (1.05, 1.15) | 1.06 (0.92, 1.20) | 1.08* (1.00, 1.17) |
| Community Cancer Program | 0.94 (0.87, 1.02) | 0.92* (0.86, 0.99) | 0.84 (0.70, 1.01) | 1.05 (0.93, 1.18) |
| Integrated Network Cancer Program | 0.99 (0.92, 1.05) | 1.03 (0.97, 1.09) | 1.08 (0.90, 1.29) | 1.09 (0.99, 1.20) |
| **Facility location** | | | | |
| West | 1 | 1 | 1 | 1 |
| South | 0.99 (0.93, 1.06) | 1.08* (1.01, 1.14) | 1.01 (0.82, 1.25) | 0.99 (0.87, 1.12) |
| Midwest | 0.99 (0.92, 1.07) | 0.97 (0.92, 1.03) | 0.92 (0.75, 1.14) | 0.93 (0.82, 1.06) |
| Northeast | 1.01 (0.92, 1.10) | 1.08* (1.02, 1.16) | 0.98 (0.78, 1.23) | 1.00 (0.88, 1.13) |
| **Charlson-Deyo score** | | | | |
| 0 | 1 | 1 | 1 | 1 |
| 1 | 1.02 (0.97, 1.09) | 1.00 (0.95, 1.05) | 1.08 (0.92, 1.27) | 1.07 (0.98, 1.18) |
| 2 | 1.11 (0.98, 1.26) | 1.04 (0.95, 1.13) | 0.72** (0.59, 0.89) | 0.95 (0.82, 1.10) |
| 3 | 1.17* (1.02, 1.35) | 0.98 (0.90, 1.07) | 0.85 (0.66, 1.09) | 1.25* (1.04, 1.50) |
| **Cancer stage** | | | | |
| I | 1 | 1 | 1 | 1 |
| II | 1.05 (0.92, 1.20) | 1.05* (1.00, 1.09) | 1.02 (0.89, 1.18) | 1.01 (0.94, 1.10) |
| III | 1.11 (0.95, 1.30) | 1.10* (1.02, 1.18) | 1.07 (0.89, 1.29) | 1.11* (1.02, 1.22) |
| IV | 0.89 (0.66, 1.21) | 0.91 (0.77, 1.07) | 0.65* (0.47, 0.92) | 1.12 (0.97, 1.28) |
| **Tumor grade** | | | | |
| I | 1 | 1 | 1 | 1 |
| II | 1.01 (0.96, 1.07) | 1.02 (0.98, 1.07) | 1.12 (0.62, 2.03) | 0.95 (0.79, 1.14) |
| III | 0.96 (0.86, 1.08) | 0.94* (0.90, 0.99) | 1.01 (0.56, 1.82) | 0.91 (0.76, 1.09) |
| IV | - | 0.63 (0.30, 1.36) | 0.88 (0.43, 1.78) | 1.04 (0.78, 1.38) |
| **Year of diagnosis** | 1.01 (0.99, 1.04) | 1.01 (0.99, 1.03) | 1.03 (0.98, 1.08) | 1.00 (0.98, 1.03) |
| **Age at diagnosis** | 0.99* (0.99, 1.00) | 0.99** (0.99, 1.00) | 1.00 (0.99, 1.01) | 1.00 (1.00, 1.00) |

Significance: * < 0.05, ** < 0.01, *** < 0.001

**-** : not estimable due to small sample size
--: infinite estimated hazard ratio

**Table S6**: Estimated hazard ratios with 95% confidence intervals from adjusted Cox models stratified by cancer stage

| **Characteristics** | **Hazard ratio with 95% confidence interval** | | | | |
| --- | --- | --- | --- | --- | --- |
|  | **Subset model: Stage 0 (n = 56,235)** | **Subset model: Stage I (n = 142,530)** | **Subset model: Stage II (n = 54,119)** | **Subset model: Stage III (n = 16,158)** | **Subset model: Stage IV (n = 5,945)** |
| **Primary treatment** | | | | | |
| Surgery | 1 | 1 | 1 | 1 | 1 |
| Chemotherapy | 1.54* (1.07, 2.22) | 1.72*** (1.5, 1.96) | 1.21*** (1.09, 1.34) | 1.23*** (1.1, 1.39) | 0.95 (0.79, 1.14) |
| Radiation | 0.58 (0.28, 1.22) | 0.70* (0.5, 0.99) | 1.25 (0.84, 1.85) | 1.63 (0.95, 2.77) | 1.81*** (1.44, 2.27) |
| Endocrine therapy | 1.29 (0.89, 1.86) | 1.49*** (1.31, 1.71) | 1.03 (0.85, 1.26) | 1.28* (1.04, 1.57) | 1.24* (1.02, 1.52) |
| **Great circle distance in miles** | | | | | |
| < 10.0 | 1 | 1 | 1 | 1 | 1 |
| 10.1-20.0 | 1.02 (0.9, 1.16) | 0.94 (0.88, 1.02) | 0.94 (0.86, 1.03) | 1.03 (0.89, 1.19) | 0.93 (0.8, 1.07) |
| > 20.0 | 0.91 (0.77, 1.08) | 0.90* (0.81, 0.99) | 0.91 (0.81, 1.02) | 0.86 (0.73, 1.01) | 0.82* (0.69, 0.99) |
| **Weeks from diagnosis to initial treatment** | | | | | |
| < 8 | 1 | 1 | 1 | 1 | 1 |
| 8 - 12 | 1.07 (0.94, 1.21) | 1.13** (1.05, 1.23) | 1.03 (0.92, 1.15) | 1.03 (0.89, 1.19) | 0.93 (0.76, 1.13) |
| > 12 | 1.25** (1.06, 1.47) | 1.27*** (1.13, 1.44) | 1.47*** (1.26, 1.70) | 1.22 (0.99, 1.50) | 0.74* (0.55, 0.99) |
| **Race and ethnicity** | | | | | |
| White, Non-Hispanic | 1 | 1 | 1 | 1 | 1 |
| Black, Non-Hispanic | 1.00 (0.85, 1.18) | 0.98 (0.87, 1.1) | 0.91 (0.79, 1.06) | 0.92 (0.76, 1.1) | 1.12 (0.91, 1.38) |
| Hispanic | 0.65 (0.39, 1.06) | 0.80 (0.59, 1.09) | 0.63** (0.44, 0.89) | 0.72 (0.49, 1.05) | 1.20 (0.83, 1.75) |
| Asian, Non-Hispanic | 0.65* (0.46, 0.93) | 0.61*** (0.48, 0.76) | 0.66** (0.51, 0.85) | 0.55** (0.38, 0.8) | 0.78 (0.51, 1.19) |
| AIAN, Non-Hispanic | 2.36** (1.3, 4.28) | 0.78 (0.5, 1.2) | 1.89** (1.25, 2.86) | 1.46 (0.66, 3.26) | 1.25 (0.59, 2.62) |
| NHPI, Non-Hispanic | 1.68 (0.49, 5.74) | 1.47* (1.03, 2.1) | 1.02 (0.52, 2.02) | 1.51 (0.55, 4.13) | 1.16 (0.61, 2.18) |
| Other | 1.00 (0.56, 1.78) | 0.50** (0.3, 0.82) | 0.91 (0.59, 1.4) | 0.73 (0.38, 1.37) | 0.74 (0.39, 1.39) |
| **Age at diagnosis** | | | | | |
| < 50 | 1 | 1 | 1 | 1 | 1 |
| 50-59 | 1.49*** (1.19, 1.86) | 1.60*** (1.37, 1.86) | 1.48*** (1.28, 1.72) | 1.22* (1.02, 1.45) | 1.12 (0.92, 1.35) |
| 60-69 | 2.68*** (2.14, 3.36) | 2.77*** (2.36, 3.24) | 2.14*** (1.84, 2.49) | 1.60*** (1.35, 1.9) | 1.09 (0.87, 1.35) |
| 70-79 | 7.02*** (5.5, 8.96) | 5.83*** (4.89, 6.95) | 4.09*** (3.36, 4.99) | 2.48*** (2, 3.08) | 1.36* (1.03, 1.79) |
| 80+ | 19.33*** (14.91, 25.07) | 17.33*** (14.31, 21) | 11.48*** (9.4, 14.01) | 6.69*** (5.29, 8.45) | 2.04*** (1.51, 2.75) |
| **Education: percent no high school degree** | | | | | |
| No HSD >=15.3% | 1 | 1 | 1 | 1 | 1 |
| No HSD 9.1% - 15.2% | 1.12 (0.94, 1.34) | 1.08 (0.97, 1.21) | 0.97 (0.85, 1.1) | 0.97 (0.81, 1.15) | 1.02 (0.84, 1.24) |
| No HSD 5.0% - 9.0% | 1.15 (0.94, 1.4) | 0.97 (0.85, 1.1) | 0.90 (0.77, 1.06) | 0.87 (0.72, 1.06) | 0.94 (0.74, 1.19) |
| No HSD < 5.0% | 0.97 (0.76, 1.22) | 0.88 (0.76, 1.01) | 0.84* (0.71, 0.99) | 0.79 (0.63, 1) | 0.89 (0.69, 1.15) |
| **Median household income** | | | | | |
| < $46,227 | 1 | 1 | 1 | 1 | 1 |
| $46,227 - $57,856 | 0.86 (0.73, 1.01) | 0.87* (0.77, 1) | 0.88 (0.77, 1.01) | 1.11 (0.9, 1.37) | 1.03 (0.81, 1.3) |
| $57,857 - $74,062 | 0.79* (0.66, 0.95) | 0.81** (0.71, 0.93) | 0.82** (0.71, 0.94) | 0.96 (0.76, 1.21) | 0.92 (0.72, 1.19) |
| >= $74,063 | 0.66*** (0.54, 0.81) | 0.76*** (0.65, 0.89) | 0.77** (0.65, 0.92) | 0.91 (0.69, 1.19) | 0.96 (0.73, 1.26) |
| **Insurance status** | | | | | |
| Private | 1 | 1 | 1 | 1 | 1 |
| Uninsured | 2.34*** (1.59, 3.45) | 1.24 (0.91, 1.69) | 1.61*** (1.24, 2.09) | 1.51** (1.14, 1.99) | 1.16 (0.78, 1.73) |
| Medicaid | 1.64*** (1.25, 2.17) | 1.88*** (1.59, 2.22) | 1.50*** (1.28, 1.75) | 1.42*** (1.19, 1.7) | 1.43*** (1.18, 1.73) |
| Medicare | 1.58*** (1.38, 1.81) | 1.45*** (1.33, 1.59) | 1.37*** (1.21, 1.54) | 1.32*** (1.12, 1.54) | 1.35** (1.12, 1.63) |
| Other Government | 1.66* (1.02, 2.7) | 1.49** (1.11, 2) | 0.87 (0.57, 1.34) | 1.13 (0.72, 1.79) | 1.06 (0.55, 2.06) |
| **Living area** | | | | | |
| Metro | 1 | 1 | 1 | 1 | 1 |
| Urban | 1.02 (0.84, 1.23) | 1.06 (0.92, 1.22) | 0.97 (0.83, 1.14) | 1.09 (0.9, 1.33) | 1.11 (0.87, 1.42) |
| Rural | 1.06 (0.73, 1.53) | 1.06 (0.83, 1.36) | 1.24 (0.91, 1.7) | 0.97 (0.65, 1.45) | 1.14 (0.77, 1.7) |
| **Facility type** | | | | | |
| Comprehensive Community Cancer Program | 1 | 1 | 1 | 1 | 1 |
| Academic/Research Program | 0.95 (0.76, 1.19) | 0.96 (0.78, 1.17) | 0.91 (0.73, 1.12) | 0.81 (0.63, 1.05) | 0.86 (0.67, 1.12) |
| Community Cancer Program | 1.41* (1.08, 1.82) | 1.38*** (1.16, 1.64) | 1.27* (1.03, 1.56) | 1.31 (1, 1.72) | 1.21 (0.88, 1.66) |
| Integrated Network Cancer Program | 1.27 (1, 1.61) | 1.16 (0.95, 1.42) | 1.16 (0.9, 1.49) | 1.20 (0.89, 1.63) | 0.98 (0.73, 1.32) |
| **Facility location** | | | | | |
| West | 1 | 1 | 1 | 1 | 1 |
| South | 1.07 (0.79, 1.45) | 0.96 (0.77, 1.21) | 0.97 (0.72, 1.3) | 0.87 (0.62, 1.22) | 0.89 (0.61, 1.29) |
| Midwest | 0.99 (0.72, 1.36) | 0.89 (0.7, 1.13) | 0.96 (0.71, 1.3) | 0.86 (0.61, 1.22) | 1.03 (0.7, 1.51) |
| Northeast | 1.20 (0.87, 1.67) | 1.07 (0.81, 1.41) | 1.03 (0.72, 1.47) | 1.12 (0.74, 1.69) | 1.22 (0.81, 1.84) |
| **Charlson-Deyo score** | | | | | |
| 0 | 1 | 1 | 1 | 1 | 1 |
| 1 | 1.50*** (1.31, 1.72) | 1.58*** (1.46, 1.71) | 1.54*** (1.41, 1.67) | 1.24** (1.08, 1.44) | 1.16 (0.96, 1.39) |
| 2 | 2.09*** (1.7, 2.56) | 2.08*** (1.8, 2.4) | 2.01*** (1.73, 2.34) | 1.74*** (1.35, 2.25) | 1.62** (1.2, 2.2) |
| 3 | 2.91*** (2.08, 4.07) | 3.52*** (2.97, 4.16) | 2.71*** (2.2, 3.34) | 1.95*** (1.44, 2.66) | 2.77*** (1.98, 3.87) |
| **Tumor grade** | | | | | |
| I | 1 | 1 | 1 | 1 | 1 |
| II | 0.99 (0.86, 1.15) | 1.12*** (1.06, 1.19) | 1.15** (1.04, 1.28) | 1.03 (0.85, 1.23) | 1.64*** (1.22, 2.2) |
| III | 0.94 (0.81, 1.1) | 1.26*** (1.16, 1.36) | 1.34*** (1.2, 1.49) | 1.19 (0.98, 1.44) | 2.30*** (1.71, 3.09) |
| IV | 1.01 (0.72, 1.43) | 1.19 (0.59, 2.43) | 0.62 (0.23, 1.69) | 1.70 (0.87, 3.34) | 4.06** (1.59, 10.35) |
| **Year of diagnosis** | 1.42*** (1.29, 1.57) | 1.43*** (1.28, 1.6) | 1.36*** (1.24, 1.5) | 1.27*** (1.15, 1.41) | 1.14*** (1.05, 1.22) |

Significance: * < 0.05, ** < 0.01, *** < 0.001

**-** : not estimable due to small sample size
--: infinite estimated hazard ratio

**Table S7**: Estimated hazard ratios with 95% confidence intervals from adjusted Cox models stratified by molecular subtype

| **Characteristics** | **Hazard ratio with 95% confidence interval** | | | |
| --- | --- | --- | --- | --- |
|  | **Luminal A (n = 3,943)** | **Luminal B (n = 7,015)** | **HER2-Enriched (n = 785)** | **Triple-Negative (n = 1,721)** |
| **Primary treatment** | | | | |
| Surgery | 1 | 1 | 1 | 1 |
| Chemotherapy | 2.04 (0.67, 6.2) | 1.23 (0.87, 1.75) | 1.32 (0.46, 3.82) | 1.09 (0.74, 1.63) |
| Radiation | 0.83 (0.23, 2.95) | 1.85 (0.96, 3.58) | 2.22 (0.2, 24.21) | 1.33 (0.14, 13.15) |
| Endocrine therapy | 0.54 (0.21, 1.42) | 1.56* (1.06, 2.29) | - | - |
| **Great circle distance in miles** | | | | |
| < 10.0 | 1 | 1 | 1 | 1 |
| 10.1-20.0 | 0.79 (0.41, 1.5) | 0.86 (0.63, 1.16) | 0.73 (0.19, 2.76) | 0.86 (0.47, 1.57) |
| > 20.0 | 0.69 (0.37, 1.3) | 0.90 (0.65, 1.26) | 0.43 (0.1, 1.81) | 1.04 (0.52, 2.07) |
| **Weeks from diagnosis to initial treatment** | | | | |
| < 8 | 1 | 1 | 1 | 1 |
| 8 - 12 | 0.84 (0.44, 1.61) | 1.04 (0.73, 1.49) | 1.46 (0.29, 7.38) | 0.95 (0.53, 1.71) |
| > 12 | 1.17 (0.47, 2.91) | 1.64 (0.99, 2.74) | 1.80 (0.37, 8.74) | 0.24 (0.03, 1.96) |
| **Race and ethnicity** | | | | |
| White, Non-Hispanic | 1 | 1 | 1 | 1 |
| Black, Non-Hispanic | 1.63 (0.86, 3.08) | 0.68 (0.44, 1.06) | 0.82 (0.25, 2.77) | 0.94 (0.61, 1.45) |
| Hispanic | 1.53 (0.2, 11.97) | 0.76 (0.28, 2.02) | - | 2.09 (0.55, 7.98) |
| Asian, Non-Hispanic | 0.77 (0.1, 6.23) | 0.68 (0.3, 1.51) | 1.24 (0.15, 10.51) | 1.44 (0.68, 3.07) |
| AIAN, Non-Hispanic | - | 0.84 (0.13, 5.54) | - | 1.92 (0.18, 21.14) |
| NHPI, Non-Hispanic | - | - | - | - |
| Other | 1.83 (0.24, 14.21) | 0.42 (0.09, 2.03) | 3.46 (0.62, 19.42) | 2.08 (0.45, 9.55) |
| **Education: percent no high school degree** | | | | |
| No HSD >=15.3% | 1 | 1 | 1 | 1 |
| No HSD 9.1% - 15.2% | 1.04 (0.54, 2.01) | 1.16 (0.78, 1.72) | 0.85 (0.36, 2.01) | 1.06 (0.57, 1.97) |
| No HSD 5.0% - 9.0% | 1.22 (0.61, 2.44) | 1.15 (0.74, 1.8) | 0.45 (0.13, 1.57) | 0.80 (0.37, 1.74) |
| No HSD < 5.0% | 0.94 (0.45, 1.94) | 0.93 (0.53, 1.62) | 0.10* (0.01, 0.78) | 0.43* (0.19, 0.95) |
| **Median household income** | | | | |
| < $46,227 | 1 | 1 | 1 | 1 |
| $46,227 - $57,856 | 0.96 (0.42, 2.17) | 0.85 (0.55, 1.32) | 0.64 (0.19, 2.14) | 1.07 (0.54, 2.11) |
| $57,857 - $74,062 | 0.75 (0.33, 1.71) | 0.76 (0.49, 1.18) | 0.84 (0.2, 3.61) | 1.28 (0.66, 2.5) |
| >= $74,063 | 0.84 (0.39, 1.79) | 0.72 (0.42, 1.22) | 0.77 (0.17, 3.57) | 1.67 (0.79, 3.52) |
| **Insurance status** | | | | |
| Private | 1 | 1 | 1 | 1 |
| Uninsured | 6.47** (1.58, 26.55) | 1.16 (0.41, 3.24) | - | 2.25 (0.59, 8.57) |
| Medicaid | 1.80 (0.68, 4.77) | 1.07 (0.62, 1.83) | 1.47 (0.36, 5.91) | 2.67* (1.25, 5.71) |
| Medicare | 0.72 (0.34, 1.53) | 1.14 (0.78, 1.65) | 0.83 (0.14, 4.87) | 1.35 (0.69, 2.61) |
| Other Government | 5.03** (1.56, 16.2) | 0.85 (0.19, 3.8) | - | 0.61 (0.09, 4.12) |
| **Living area** | | | | |
| Metro | 1 | 1 | 1 | 1 |
| Urban | 1.21 (0.65, 2.23) | 0.89 (0.58, 1.38) | 0.65 (0.12, 3.69) | 1.24 (0.48, 3.18) |
| Rural | - | 1.00 (0.33, 3.02) | - | 0.66 (0.17, 2.5) |
| **Facility type** | | | | |
| Comprehensive Community Cancer Program | 1 | 1 | 1 | 1 |
| Academic/Research Program | 0.74 (0.39, 1.42) | 0.88 (0.63, 1.22) | 1.91 (0.67, 5.43) | 0.90 (0.57, 1.4) |
| Community Cancer Program | 1.46 (0.71, 3.01) | 1.37 (0.86, 2.19) | 0.71 (0.13, 3.85) | 0.81 (0.28, 2.39) |
| Integrated Network Cancer Program | 0.46 (0.21, 1.04) | 1.02 (0.69, 1.5) | 1.02 (0.3, 3.42) | 0.71 (0.38, 1.33) |
| **Facility location** | | | | |
| West | 1 | 1 | 1 | 1 |
| South | 1.48 (0.68, 3.25) | 1.05 (0.66, 1.67) | 1.39 (0.28, 6.98) | 0.97 (0.34, 2.78) |
| Midwest | 1.94 (0.81, 4.65) | 1.04 (0.64, 1.71) | 1.64 (0.31, 8.55) | 1.11 (0.39, 3.13) |
| Northeast | 2.04 (0.82, 5.04) | 0.91 (0.54, 1.56) | 0.80 (0.17, 3.77) | 1.07 (0.39, 2.97) |
| **Charlson-Deyo score** | | | | |
| 0 | 1 | 1 | 1 | 1 |
| 1 | 1.09 (0.58, 2.04) | 1.19 (0.83, 1.72) | 0.65 (0.18, 2.32) | 1.42 (0.8, 2.5) |
| 2 | 1.57 (0.58, 4.24) | 2.02** (1.27, 3.22) | 0.55 (0.12, 2.47) | 1.46 (0.54, 3.96) |
| 3 | 2.66* (1.08, 6.58) | 3.01*** (1.82, 4.96) | 1.14 (0.11, 11.52) | 1.94 (0.63, 5.92) |
| **Cancer stage** | | | | |
| I | 1 | 1 | 1 | 1 |
| II | 1.71 (0.74, 3.95) | 1.78*** (1.27, 2.49) | 0.77 (0.29, 2.05) | 1.46 (0.82, 2.58) |
| III | 2.84 (0.72, 11.14) | 4.12*** (2.8, 6.07) | 2.07 (0.64, 6.7) | 4.35 (2.43, 7.78) |
| IV | 9.07*** (3.43, 24.01) | 7.90*** (5.15, 12.13) | 8.80** (1.71, 45.43) | 17.16 (9, 32.72) |
| **Tumor grade** | | | | |
| I | 1 | 1 | 1 | 1 |
| II | 0.92 (0.56, 1.51) | 1.24 (0.82, 1.87) | -- | 0.82 (0.16, 4.13) |
| III | 1.83 (0.61, 5.5) | 1.55* (1, 2.4) | -- | 1.49 (0.32, 7.01) |
| IV | - | - | 5.68 (0.39, 82.28) | - |
| **Year of diagnosis** | 2.15*** (1.54, 3.02) | 1.95*** (1.61, 2.38) | 2.26** (1.22, 4.21) | 1.74*** (1.28, 2.36) |
| **Age at diagnosis** | 1.10*** (1.07, 1.14) | 1.04*** (1.02, 1.06) | 1.06 (0.99, 1.14) | 1.04** (1.01, 1.06) |

Significance: * < 0.05, ** < 0.01, *** < 0.001

**-** : not estimable due to small sample size
--: infinite estimated hazard ratio

**Table S8**: Estimated rate ratios with 95% confidence intervals from adjusted Poisson regression models via generalized estimating equations stratified by primary treatment

| **Characteristics** | **Rate ratio with 95% confidence interval** | | | |
| --- | --- | --- | --- | --- |
|  | **Surgery (n = 231,795)** | **Chemotherapy (n = 30,902)** | **Radiation (n = 2,745)** | **Endocrine therapy (n = 7,546)** |
| **Great circle distance in miles** | | | | |
| < 10.0 | 1 | 1 | 1 | 1 |
| 10.1-20.0 | 1.01* (1.00, 1.02) | 1.00 (0.98, 1.03) | 1.02 (0.91, 1.15) | 1.02 (0.96, 1.08) |
| > 20.0 | 1.05*** (1.04, 1.06) | 1.01 (0.99, 1.04) | 1.02 (0.93, 1.12) | 1.02 (0.96, 1.08) |
| **Race and ethnicity** | | | | |
| White, Non-Hispanic | 1 | 1 | 1 | 1 |
| Black, Non-Hispanic | 1.18*** (1.16, 1.19) | 1.19*** (1.13, 1.24) | 1.24** (1.08, 1.43) | 1.19*** (1.10, 1.29) |
| Hispanic | 1.13*** (1.09, 1.18) | 1.18*** (1.11, 1.25) | 1.25 (0.81, 1.94) | 1.17 (0.97, 1.40) |
| Asian, Non-Hispanic | 1.03*** (1.01, 1.05) | 1.01 (0.98, 1.05) | 1.11 (0.96, 1.28) | 1.14 (0.99, 1.32) |
| AIAN, Non-Hispanic | 1.13** (1.05, 1.21) | 1.08 (0.97, 1.20) | 1.30 (0.79, 2.13) | 1.47 (0.89, 2.45) |
| NHPI, Non-Hispanic | 1.02 (0.96, 1.09) | 1.08 (0.95, 1.22) | 0.65 (0.36, 1.17) | 1.04 (0.65, 1.66) |
| Other | 1.11*** (1.07, 1.16) | 1.08* (1.01, 1.15) | 1.52 (0.90, 2.57) | 1.10 (0.92, 1.32) |
| **Age at diagnosis** | | | | |
| < 50 | 1 | 1 | 1 | 1 |
| 50-59 | 0.93*** (0.92, 0.94) | 1.04** (1.01, 1.07) | 0.87* (0.76, 1.00) | 1.03 (0.95, 1.11) |
| 60-69 | 0.90*** (0.89, 0.91) | 1.05** (1.02, 1.07) | 0.84* (0.73, 0.97) | 0.94 (0.86, 1.02) |
| 70-79 | 0.86*** (0.85, 0.88) | 1.03 (0.98, 1.08) | 0.80** (0.68, 0.94) | 0.92 (0.82, 1.03) |
| 80+ | 0.85*** (0.83, 0.86) | 1.07 (0.96, 1.18) | 0.79* (0.64, 0.97) | 0.83*** (0.74, 0.93) |
| **Education: percent no high school degree** | | | | |
| No HSD >=15.3% | 1 | 1 | 1 | 1 |
| No HSD 9.1% - 15.2% | 0.97*** (0.96, 0.98) | 0.96** (0.94, 0.99) | 0.89 (0.77, 1.03) | 0.95 (0.88, 1.03) |
| No HSD 5.0% - 9.0% | 0.96*** (0.95, 0.97) | 0.94** (0.90, 0.97) | 0.84* (0.72, 0.97) | 0.92 (0.84, 1.00) |
| No HSD < 5.0% | 0.95*** (0.94, 0.97) | 0.90*** (0.87, 0.93) | 0.86 (0.74, 1.01) | 0.89* (0.80, 0.97) |
| **Median household income** | | | | |
| < $46,227 | 1 | 1 | 1 | 1 |
| $46,227 - $57,856 | 1.01* (1.00, 1.03) | 1.01 (0.98, 1.03) | 0.96 (0.80, 1.16) | 1.01 (0.92, 1.11) |
| $57,857 - $74,062 | 1.01 (0.99, 1.02) | 1.01 (0.97, 1.05) | 0.92 (0.78, 1.09) | 0.93 (0.85, 1.01) |
| >= $74,063 | 1.01 (1.00, 1.03) | 1.00 (0.97, 1.04) | 0.90 (0.76, 1.06) | 0.92 (0.84, 1.02) |
| **Insurance status** | | | | |
| Private | 1 | 1 | 1 | 1 |
| Uninsured | 1.17*** (1.13, 1.21) | 1.28*** (1.22, 1.34) | 0.79 (0.49, 1.28) | 1.30*** (1.12, 1.50) |
| Medicaid | 1.15*** (1.13, 1.17) | 1.19*** (1.16, 1.22) | 1.08 (0.89, 1.31) | 1.08 (0.97, 1.21) |
| Medicare | 1.02*** (1.01, 1.03) | 1.09** (1.03, 1.16) | 1.03 (0.94, 1.12) | 0.97 (0.89, 1.05) |
| Other Government | 1.07*** (1.04, 1.11) | 1.10** (1.04, 1.17) | 1.02 (0.62, 1.68) | 0.99 (0.82, 1.19) |
| **Living area** | | | | |
| Metro | 1 | 1 | 1 | 1 |
| Urban | 0.95*** (0.93, 0.96) | 1.00 (0.97, 1.02) | 0.96 (0.84, 1.08) | 1.03 (0.94, 1.13) |
| Rural | 0.89*** (0.87, 0.91) | 0.94* (0.89, 1.00) | 0.86 (0.69, 1.07) | 0.96 (0.81, 1.13) |
| **Facility type** | | | | |
| Comprehensive Community Cancer Program | 1 | 1 | 1 | 1 |
| Academic/Research Program | 1.16*** (1.15, 1.17) | 1.08*** (1.06, 1.11) | 1.00 (0.91, 1.09) | 1.11*** (1.05, 1.17) |
| Community Cancer Program | 0.98* (0.97, 1.00) | 0.99 (0.96, 1.02) | 0.99 (0.84, 1.16) | 1.03 (0.92, 1.15) |
| Integrated Network Cancer Program | 1.08*** (1.06, 1.09) | 1.00 (0.97, 1.04) | 0.95 (0.85, 1.06) | 1.15*** (1.07, 1.24) |
| **Facility location** | | | | |
| West | 1 | 1 | 1 | 1 |
| South | 0.97*** (0.96, 0.98) | 0.97 (0.94, 1.00) | 0.90 (0.81, 1.00) | 0.90** (0.83, 0.97) |
| Midwest | 0.89*** (0.88, 0.90) | 0.88*** (0.85, 0.91) | 0.86** (0.77, 0.95) | 0.87** (0.80, 0.95) |
| Northeast | 1.02** (1.01, 1.03) | 1.01 (0.98, 1.05) | 1.10 (0.97, 1.25) | 1.02 (0.93, 1.10) |
| **Charlson-Deyo score** | | | | |
| 0 | 1 | 1 | 1 | 1 |
| 1 | 1.03*** (1.02, 1.04) | 1.02 (0.99, 1.05) | 0.87** (0.80, 0.95) | 1.03 (0.96, 1.11) |
| 2 | 1.05*** (1.03, 1.07) | 1.01 (0.97, 1.06) | 0.96 (0.78, 1.17) | 1.08 (0.97, 1.21) |
| 3 | 1.10*** (1.07, 1.13) | 1.08* (1.01, 1.16) | 1.20 (0.80, 1.80) | 1.05 (0.93, 1.19) |
| **Cancer stage** | | | | |
| 0 | 1 | 1 | 1 | 1 |
| I | 0.92*** (0.91, 0.93) | 1.13*** (1.08, 1.18) | 0.91 (0.81, 1.01) | 0.91 (0.83, 1.01) |
| II | 0.96*** (0.94, 0.97) | 1.10*** (1.05, 1.15) | 1.37*** (1.15, 1.63) | 0.91 (0.81, 1.01) |
| III | 1.00 (0.98, 1.03) | 1.06* (1.01, 1.11) | 1.75*** (1.35, 2.27) | 0.81*** (0.72, 0.91) |
| IV | 0.92 (0.83, 1.02) | 1.02 (0.97, 1.07) | 0.55*** (0.45, 0.68) | 0.70*** (0.63, 0.77) |
| **Tumor grade** | | | | |
| I | 1 | 1 | 1 | 1 |
| II | 1.04*** (1.03, 1.05) | 0.92 (0.82, 1.03) | 0.96 (0.90, 1.03) | 1.02 (0.96, 1.09) |
| III | 1.01 (0.99, 1.02) | 0.85** (0.76, 0.95) | 1.04 (0.93, 1.17) | 1.01 (0.92, 1.10) |
| IV | 1.08* (1.01, 1.14) | 0.91 (0.73, 1.12) | 0.85 (0.60, 1.21) | 0.87 (0.59, 1.28) |
| **Year of diagnosis** | 1.03*** (1.02, 1.03) | 1.01*** (1, 1.01) | 1.00 (0.98, 1.01) | 1.00 (0.99, 1.01) |

Significance: * < 0.05, ** < 0.01, *** < 0.001

**Table S9**: Estimated hazard ratios with 95% confidence intervals from adjusted Cox models stratified by primary treatment

| **Characteristics** | **Hazard ratio with 95% confidence interval** | | | |
| --- | --- | --- | --- | --- |
|  | **Subset model: Surgery (n = 231,795)** | **Subset model: Chemotherapy (n = 30,902)** | **Subset model: Radiation (n = 2,745)** | **Subset model: Endocrine therapy (n = 7,546)** |
| **Great circle distance in miles** | | | | |
| < 10.0 | 1 | 1 | 1 | 1 |
| 10.1-20.0 | 0.96 (0.9, 1.03) | 0.92 (0.81, 1.03) | 1.30 (0.95, 1.79) | 0.83 (0.66, 1.04) |
| > 20.0 | 0.89* (0.81, 0.98) | 0.86* (0.75, 0.99) | 0.99 (0.68, 1.44) | 0.84 (0.65, 1.08) |
| **Weeks from diagnosis to initial treatment** | | | | |
| < 8 | 1 | 1 | 1 | 1 |
| 8 - 12 | 1.09** (1.02, 1.16) | 0.98 (0.85, 1.13) | 1.04 (0.67, 1.62) | 0.82 (0.63, 1.07) |
| > 12 | 1.31*** (1.19, 1.43) | 1.14 (0.91, 1.42) | 0.78 (0.50, 1.22) | 1.32 (0.96, 1.83) |
| **Race and ethnicity** | | | | |
| White, Non-Hispanic | 1 | 1 | 1 | 1 |
| Black, Non-Hispanic | 0.94 (0.85, 1.05) | 1.01 (0.88, 1.16) | 1.12 (0.7, 1.79) | 0.92 (0.71, 1.19) |
| Hispanic | 0.70** (0.55, 0.89) | 0.93 (0.7, 1.23) | 1.5 (0.39, 5.74) | 0.95 (0.52, 1.73) |
| Asian, Non-Hispanic | 0.60*** (0.5, 0.71) | 0.62** (0.45, 0.86) | 0.71 (0.28, 1.81) | 1.15 (0.78, 1.7) |
| AIAN, Non-Hispanic | 1.29 (0.96, 1.75) | 1.47 (0.87, 2.5) | 1.99 (0.17, 23.74) | 0.74 (0.22, 2.44) |
| NHPI, Non-Hispanic | 1.07 (0.82, 1.41) | 3.67*** (2.24, 6.01) | 1.68 (0.33, 8.52) | 0.45 (0.06, 3.16) |
| Other | 0.68* (0.5, 0.94) | 0.75 (0.45, 1.27) | 0.77 (0.22, 2.66) | 1.67 (0.79, 3.51) |
| **Age at diagnosis** | | | | |
| < 50 | 1 | 1 | 1 | 1 |
| 50-59 | 1.53*** (1.39, 1.68) | 1.22** (1.07, 1.38) | 0.86 (0.53, 1.39) | 1.26 (0.89, 1.77) |
| 60-69 | 2.47*** (2.23, 2.73) | 1.49*** (1.29, 1.72) | 1.03 (0.64, 1.66) | 1.57** (1.13, 2.17) |
| 70-79 | 5.32*** (4.69, 6.03) | 2.03*** (1.67, 2.47) | 1.12 (0.64, 1.96) | 2.32*** (1.57, 3.42) |
| 80+ | 15.31*** (13.38, 17.53) | 3.61*** (2.79, 4.66) | 3.26*** (1.76, 6.04) | 5.35*** (3.61, 7.93) |
| **Education: percent no high school degree** | | | | |
| No HSD >=15.3% | 1 | 1 | 1 | 1 |
| No HSD 9.1% - 15.2% | 1.03 (0.92, 1.15) | 1.01 (0.88, 1.16) | 1.02 (0.68, 1.51) | 0.97 (0.74, 1.29) |
| No HSD 5.0% - 9.0% | 0.94 (0.83, 1.06) | 0.93 (0.78, 1.11) | 0.86 (0.53, 1.4) | 1.07 (0.81, 1.42) |
| No HSD < 5.0% | 0.87* (0.75, 0.99) | 0.79* (0.65, 0.94) | 0.68 (0.42, 1.1) | 0.94 (0.68, 1.3) |
| **Median household income** | | | | |
| < $46,227 | 1 | 1 | 1 | 1 |
| $46,227 - $57,856 | 0.89 (0.79, 1.02) | 0.98 (0.84, 1.13) | 0.66 (0.41, 1.05) | 1.07 (0.8, 1.42) |
| $57,857 - $74,062 | 0.82** (0.72, 0.94) | 0.87 (0.74, 1.03) | 0.63 (0.38, 1.04) | 0.99 (0.73, 1.34) |
| >= $74,063 | 0.76*** (0.65, 0.88) | 0.90 (0.74, 1.09) | 0.74 (0.45, 1.23) | 0.89 (0.63, 1.24) |
| **Insurance status** | | | | |
| Private | 1 | 1 | 1 | 1 |
| Uninsured | 1.54*** (1.27, 1.85) | 1.23 (0.97, 1.57) | 2.39* (1.06, 5.42) | 1.86** (1.24, 2.78) |
| Medicaid | 1.62*** (1.44, 1.83) | 1.62*** (1.4, 1.88) | 1.61* (1.01, 2.56) | 1.23 (0.87, 1.73) |
| Medicare | 1.45*** (1.34, 1.57) | 1.38*** (1.2, 1.59) | 1.44 (0.97, 2.13) | 1.26 (0.98, 1.61) |
| Other Government | 1.25 (0.99, 1.58) | 0.91 (0.56, 1.48) | 4.84*** (2.09, 11.22) | 1.33 (0.63, 2.84) |
| **Living area** | | | | |
| Metro | 1 | 1 | 1 | 1 |
| Urban | 1.07 (0.93, 1.22) | 0.88 (0.73, 1.06) | 0.97 (0.62, 1.51) | 1.22 (0.93, 1.61) |
| Rural | 1.14 (0.93, 1.4) | 1.03 (0.73, 1.45) | 1.30 (0.32, 5.24) | 1.16 (0.66, 2.04) |
| **Facility type** | | | | |
| Comprehensive Community Cancer Program | 1 | 1 | 1 | 1 |
| Academic/Research Program | 0.93 (0.76, 1.15) | 0.81* (0.67, 0.99) | 0.70 (0.48, 1.01) | 0.94 (0.71, 1.24) |
| Community Cancer Program | 1.35** (1.12, 1.63) | 1.26 (1, 1.6) | 1.05 (0.58, 1.92) | 1.19 (0.86, 1.63) |
| Integrated Network Cancer Program | 1.23 (0.98, 1.54) | 0.90 (0.71, 1.15) | 0.99 (0.67, 1.44) | 0.95 (0.73, 1.24) |
| **Facility location** | | | | |
| West | 1 | 1 | 1 | 1 |
| South | 0.91 (0.69, 1.19) | 1.18 (0.87, 1.6) | 0.85 (0.49, 1.49) | 1.29 (0.92, 1.79) |
| Midwest | 0.87 (0.66, 1.16) | 1.16 (0.87, 1.56) | 1.43 (0.82, 2.49) | 0.98 (0.69, 1.38) |
| Northeast | 1.04 (0.75, 1.43) | 1.29 (0.93, 1.79) | 1.30 (0.72, 2.34) | 1.38 (0.94, 2.03) |
| **Charlson-Deyo score** | | | | |
| 0 | 1 | 1 | 1 | 1 |
| 1 | 1.53*** (1.43, 1.63) | 1.21** (1.06, 1.38) | 1.63** (1.14, 2.32) | 1.51*** (1.22, 1.86) |
| 2 | 2.06*** (1.85, 2.28) | 1.69*** (1.35, 2.12) | 1.99* (1.08, 3.68) | 1.92*** (1.34, 2.74) |
| 3 | 2.99*** (2.59, 3.45) | 2.62*** (2.05, 3.35) | 2.21* (1.08, 4.52) | 2.96*** (1.9, 4.62) |
| **Cancer stage** | | | | |
| 0 | 1 | 1 | 1 | 1 |
| I | 1.24*** (1.17, 1.31) | 1.45* (1, 2.11) | 1.81 (0.8, 4.07) | 1.66** (1.16, 2.4) |
| II | 1.78*** (1.67, 1.9) | 1.85*** (1.3, 2.65) | 4.70*** (2.02, 10.94) | 1.75** (1.16, 2.62) |
| III | 3.02*** (2.75, 3.33) | 3.77*** (2.63, 5.41) | 9.95*** (3.9, 25.38) | 3.56*** (2.36, 5.36) |
| IV | 9.25*** (7.32, 11.68) | 9.93*** (6.79, 14.51) | 31.35*** (14.12, 69.64) | 11.92*** (8.19, 17.33) |
| **Tumor grade** | | | | |
| I | 1 | 1 | 1 | 1 |
| II | 1.12*** (1.07, 1.18) | 1.05 (0.84, 1.31) | 1.12 (0.75, 1.67) | 1.18 (0.94, 1.49) |
| III | 1.26*** (1.19, 1.34) | 1.17 (0.94, 1.46) | 1.93** (1.27, 2.93) | 1.67*** (1.31, 2.14) |
| IV | 1.18 (0.9, 1.55) | 1.73 (0.88, 3.4) | 3.58 (0.69, 18.52) | 3.22 (0.78, 13.27) |
| **Year of diagnosis** | 1.36*** (1.23, 1.5) | 1.30*** (1.18, 1.43) | 1.11* (1.02, 1.2) | 1.29*** (1.14, 1.47) |

Significance: * < 0.05, ** < 0.01, *** < 0.001

**-** : not estimable due to small sample size
--: infinite estimated hazard ratio

**Table S10**: Estimated rate ratios with 95% confidence intervals from adjusted Poisson regression models via generalized estimating equations stratified by living area

| **Characteristics** | **Rate ratio with 95% confidence interval** | | |
| --- | --- | --- | --- |
|  | **Metro (n = 240,968)** | **Urban (n = 31,432)** | **Rural (n = 3,952)** |
| **Primary treatment** | | | |
| Surgery | 1 | 1 | 1 |
| Chemotherapy | 0.87*** (0.86, 0.88) | 0.93*** (0.90, 0.96) | 0.94 (0.87, 1.01) |
| Radiation | 1.15*** (1.11, 1.20) | 1.20*** (1.10, 1.31) | 1.18* (1.02, 1.37) |
| Endocrine therapy | 0.89*** (0.87, 0.92) | 0.96 (0.89, 1.04) | 0.95 (0.81, 1.11) |
| **Great circle distance in miles** | | | |
| < 10.0 | 1 | 1 | 1 |
| 10.1-20.0 | 1.01** (1.00, 1.02) | 1.00 (0.96, 1.03) | 1.05 (0.88, 1.24) |
| > 20.0 | 1.05*** (1.04, 1.06) | 1.02 (0.99, 1.05) | 1.11 (0.96, 1.28) |
| **Race and ethnicity** | | | |
| White, Non-Hispanic | 1 | 1 | 1 |
| Black, Non-Hispanic | 1.18*** (1.16, 1.19) | 1.17*** (1.12, 1.22) | 1.12* (1.02, 1.24) |
| Hispanic | 1.14*** (1.10, 1.18) | 1.03 (0.90, 1.17) | 1.03 (0.53, 2.03) |
| Asian, Non-Hispanic | 1.03*** (1.02, 1.05) | 1.05 (0.95, 1.17) | 0.83 (0.43, 1.58) |
| AIAN, Non-Hispanic | 1.12** (1.03, 1.21) | 1.16* (1.01, 1.33) | 1.28* (1.06, 1.55) |
| NHPI, Non-Hispanic | 1.00 (0.94, 1.07) | 1.29* (1.06, 1.56) | 0.68*** (0.61, 0.75) |
| Other | 1.11*** (1.06, 1.15) | 1.12 (0.94, 1.33) | 1.60 (0.86, 2.98) |
| **Age at diagnosis** | | | |
| < 50 | 1 | 1 | 1 |
| 50-59 | 0.95*** (0.94, 0.96) | 0.95*** (0.92, 0.98) | 1.00 (0.92, 1.08) |
| 60-69 | 0.92*** (0.91, 0.93) | 0.92*** (0.89, 0.94) | 0.90* (0.83, 0.98) |
| 70-79 | 0.88*** (0.87, 0.89) | 0.89*** (0.86, 0.93) | 0.88* (0.80, 0.97) |
| 80+ | 0.85*** (0.84, 0.87) | 0.91*** (0.86, 0.96) | 0.94 (0.83, 1.07) |
| **Education: percent no high school degree** | | | |
| No HSD >=15.3% | 1 | 1 | 1 |
| No HSD 9.1% - 15.2% | 0.95*** (0.94, 0.96) | 1.05*** (1.02, 1.08) | 1.08* (1.01, 1.15) |
| No HSD 5.0% - 9.0% | 0.94*** (0.93, 0.95) | 1.03 (1.00, 1.06) | 1.02 (0.95, 1.10) |
| No HSD < 5.0% | 0.93*** (0.91, 0.94) | 1.00 (0.96, 1.05) | 1.06 (0.97, 1.16) |
| **Median household income** | | | |
| < $46,227 | 1 | 1 | 1 |
| $46,227 - $57,856 | 1.00 (0.99, 1.02) | 1.02 (0.99, 1.05) | 1.04 (0.98, 1.11) |
| $57,857 - $74,062 | 1.00 (0.98, 1.01) | 1.01 (0.98, 1.04) | 1.07 (1.00, 1.14) |
| >= $74,063 | 1.01 (0.99, 1.02) | 1.02 (0.98, 1.07) | 1.12 (0.99, 1.27) |
| **Insurance status** | | | |
| Private | 1 | 1 | 1 |
| Uninsured | 1.21*** (1.17, 1.24) | 1.10* (1.02, 1.19) | 1.02 (0.84, 1.25) |
| Medicaid | 1.16*** (1.14, 1.18) | 1.13*** (1.08, 1.18) | 1.20** (1.07, 1.33) |
| Medicare | 1.02*** (1.01, 1.03) | 1.02 (0.99, 1.05) | 1.01 (0.94, 1.08) |
| Other Government | 1.07*** (1.03, 1.10) | 1.08 (1.00, 1.17) | 1.09 (0.90, 1.32) |
| **Facility type** | | | |
| Comprehensive Community Cancer Program | 1 | 1 | 1 |
| Academic/Research Program | 1.15*** (1.14, 1.16) | 1.20*** (1.17, 1.23) | 1.09** (1.03, 1.17) |
| Community Cancer Program | 0.99 (0.97, 1.00) | 0.96** (0.93, 0.98) | 0.92* (0.85, 1.00) |
| Integrated Network Cancer Program | 1.07*** (1.06, 1.09) | 1.04** (1.01, 1.08) | 1.05 (0.97, 1.13) |
| **Facility location** | | | |
| West | 1 | 1 | 1 |
| South | 0.98*** (0.97, 0.99) | 0.91*** (0.87, 0.95) | 1.06 (0.97, 1.16) |
| Midwest | 0.89*** (0.88, 0.90) | 0.85*** (0.82, 0.89) | 0.91* (0.84, 0.99) |
| Northeast | 1.02*** (1.01, 1.03) | 1.02 (0.97, 1.07) | 1.07 (0.91, 1.25) |
| **Charlson-Deyo score** | | | |
| 0 | 1 | 1 | 1 |
| 1 | 1.02*** (1.01, 1.03) | 1.04* (1.01, 1.08) | 1.03 (0.97, 1.10) |
| 2 | 1.04*** (1.02, 1.06) | 1.07** (1.02, 1.12) | 1.08 (0.94, 1.25) |
| 3 | 1.09*** (1.06, 1.13) | 1.09* (1.02, 1.17) | 1.24* (1.02, 1.52) |
| **Cancer stage** | | | |
| 0 | 1 | 1 | 1 |
| I | 0.93*** (0.92, 0.94) | 0.93*** (0.90, 0.96) | 0.92* (0.86, 1.00) |
| II | 0.96*** (0.95, 0.97) | 0.96* (0.93, 1.00) | 0.91* (0.83, 0.99) |
| III | 0.97*** (0.95, 0.99) | 0.97 (0.92, 1.02) | 0.99 (0.88, 1.12) |
| IV | 0.91** (0.85, 0.97) | 0.90 (0.77, 1.05) | 0.89 (0.70, 1.13) |
| **Tumor grade** | | | |
| I | 1 | 1 | 1 |
| II | 1.04*** (1.03, 1.05) | 1.02 (0.99, 1.05) | 1.02 (0.96, 1.09) |
| III | 1.00 (0.99, 1.01) | 0.96* (0.94, 0.99) | 0.99 (0.93, 1.06) |
| IV | 1.08** (1.02, 1.15) | 0.91 (0.81, 1.03) | 0.77 (0.53, 1.12) |
| **Year of diagnosis** | 1.02*** (1.02, 1.02) | 1.03*** (1.03, 1.03) | 1.02*** (1.02, 1.03) |

Significance: * < 0.05, ** < 0.01, *** < 0.001

**Table S11**: Estimated rate ratios with 95% confidence intervals from adjusted Poisson regression models via generalized estimating equations stratified by facility type

| **Characteristics** | **Rate ratio with 95% confidence interval** | | | |
| --- | --- | --- | --- | --- |
|  | **Comprehensive Community  Cancer Program (n = 121,650)** | **Academic/Research  Program (n = 90,811)** | **Community Cancer  Program (n = 20,677)** | **Integrated Network  Cancer Program (n = 50,028)** |
| **Primary treatment** | | | | |
| Surgery | 1 | 1 | 1 | 1 |
| Chemotherapy | 0.91*** (0.90, 0.93) | 0.84*** (0.82, 0.85) | 0.92*** (0.88, 0.96) | 0.84*** (0.81, 0.86) |
| Radiation | 1.24*** (1.17, 1.31) | 1.11*** (1.06, 1.18) | 1.18* (1.02, 1.36) | 1.06 (0.98, 1.16) |
| Endocrine therapy | 0.89*** (0.86, 0.93) | 0.87*** (0.84, 0.91) | 0.92 (0.82, 1.03) | 0.95 (0.89, 1.01) |
| **Great circle distance in miles** | | | | |
| < 10.0 | 1 | 1 | 1 | 1 |
| 10.1-20.0 | 1.01 (1.00, 1.02) | 1.02* (1.00, 1.03) | 1.01 (0.98, 1.04) | 1.02 (1.00, 1.05) |
| > 20.0 | 1.04*** (1.02, 1.06) | 1.07*** (1.06, 1.09) | 1.00 (0.97, 1.04) | 1.00 (0.98, 1.03) |
| **Race and ethnicity** | | | | |
| White, Non-Hispanic | 1 | 1 | 1 | 1 |
| Black, Non-Hispanic | 1.16*** (1.14, 1.19) | 1.16*** (1.14, 1.19) | 1.23*** (1.18, 1.29) | 1.17*** (1.13, 1.20) |
| Hispanic | 1.07* (1.01, 1.12) | 1.11*** (1.06, 1.16) | 1.16* (1.03, 1.30) | 1.27*** (1.11, 1.44) |
| Asian, Non-Hispanic | 1.03* (1.01, 1.06) | 1.01 (0.98, 1.04) | 1.14*** (1.06, 1.22) | 1.05 (0.99, 1.11) |
| AIAN, Non-Hispanic | 1.19*** (1.10, 1.29) | 1.17 (0.98, 1.39) | 1.07 (0.88, 1.29) | 0.93 (0.80, 1.07) |
| NHPI, Non-Hispanic | 1.02 (0.92, 1.14) | 0.99 (0.91, 1.07) | 1.49*** (1.18, 1.87) | 0.91 (0.76, 1.09) |
| Other | 1.12*** (1.05, 1.18) | 1.11*** (1.05, 1.18) | 0.97 (0.81, 1.15) | 1.13* (1.02, 1.24) |
| **Age at diagnosis** | | | | |
| < 50 | 1 | 1 | 1 | 1 |
| 50-59 | 0.95*** (0.93, 0.96) | 0.94*** (0.93, 0.96) | 0.99 (0.96, 1.03) | 0.95*** (0.92, 0.98) |
| 60-69 | 0.91*** (0.89, 0.92) | 0.92*** (0.90, 0.94) | 0.95** (0.91, 0.98) | 0.93*** (0.90, 0.96) |
| 70-79 | 0.88*** (0.87, 0.90) | 0.87*** (0.85, 0.89) | 0.90*** (0.86, 0.95) | 0.88*** (0.85, 0.91) |
| 80+ | 0.87*** (0.85, 0.89) | 0.84*** (0.81, 0.86) | 0.94 (0.88, 1.00) | 0.85*** (0.81, 0.88) |
| **Education: percent no high school degree** | | | | |
| No HSD >=15.3% | 1 | 1 | 1 | 1 |
| No HSD 9.1% - 15.2% | 0.97*** (0.95, 0.98) | 0.97** (0.95, 0.99) | 1.01 (0.97, 1.05) | 0.98 (0.94, 1.01) |
| No HSD 5.0% - 9.0% | 0.95*** (0.93, 0.97) | 0.96*** (0.94, 0.98) | 1.02 (0.98, 1.07) | 0.97 (0.93, 1.01) |
| No HSD < 5.0% | 0.95*** (0.93, 0.97) | 0.95*** (0.93, 0.97) | 0.99 (0.94, 1.03) | 0.95* (0.91, 0.99) |
| **Median household income** | | | | |
| < $46,227 | 1 | 1 | 1 | 1 |
| $46,227 - $57,856 | 1.03** (1.01, 1.05) | 0.99 (0.97, 1.02) | 0.98 (0.94, 1.03) | 0.99 (0.96, 1.02) |
| $57,857 - $74,062 | 1.03** (1.01, 1.04) | 0.97** (0.94, 0.99) | 0.98 (0.93, 1.03) | 0.98 (0.95, 1.02) |
| >= $74,063 | 1.04*** (1.02, 1.06) | 0.94*** (0.92, 0.96) | 0.97 (0.92, 1.02) | 0.98 (0.95, 1.02) |
| **Insurance status** | | | | |
| Private | 1 | 1 | 1 | 1 |
| Uninsured | 1.11*** (1.05, 1.16) | 1.23*** (1.17, 1.29) | 1.15** (1.05, 1.26) | 1.13** (1.04, 1.23) |
| Medicaid | 1.18*** (1.15, 1.21) | 1.15*** (1.12, 1.18) | 1.10*** (1.05, 1.15) | 1.13*** (1.09, 1.17) |
| Medicare | 1.03*** (1.01, 1.04) | 1.02** (1.01, 1.04) | 1.03 (1.00, 1.07) | 1.01 (0.98, 1.04) |
| Other Government | 1.09*** (1.05, 1.14) | 1.10*** (1.04, 1.16) | 1.00 (0.91, 1.11) | 1.03 (0.95, 1.10) |
| **Living area** | | | | |
| Metro | 1 | 1 | 1 | 1 |
| Urban | 0.97*** (0.95, 0.98) | 0.97** (0.94, 0.99) | 0.95*** (0.92, 0.98) | 0.97 (0.94, 1.00) |
| Rural | 0.94*** (0.90, 0.97) | 0.86*** (0.82, 0.91) | 0.89** (0.82, 0.97) | 0.91** (0.86, 0.97) |
| **Facility location** | | | | |
| West | 1 | 1 | 1 | 1 |
| South | 0.97*** (0.96, 0.99) | 0.92*** (0.89, 0.94) | 0.92*** (0.88, 0.97) | 0.97 (0.93, 1.02) |
| Midwest | 0.92*** (0.90, 0.93) | 0.85*** (0.83, 0.87) | 0.94** (0.90, 0.98) | 0.85*** (0.81, 0.89) |
| Northeast | 1.08*** (1.06, 1.10) | 0.96** (0.94, 0.99) | 1.02 (0.97, 1.07) | 0.95 (0.91, 1.00) |
| **Cancer stage** | | | | |
| Stage 0 | 1 | 1 | 1 | 1 |
| Stage I | 0.93*** (0.92, 0.95) | 0.92*** (0.91, 0.94) | 0.93*** (0.90, 0.97) | 0.92*** (0.90, 0.95) |
| Stage II | 0.96*** (0.94, 0.98) | 0.95*** (0.93, 0.97) | 1.01 (0.97, 1.06) | 0.96 (0.93, 1.00) |
| Stage III | 0.96** (0.94, 0.99) | 0.96** (0.93, 0.99) | 1.04 (0.98, 1.11) | 0.98 (0.94, 1.02) |
| Stage IV | 0.88** (0.81, 0.96) | 0.90 (0.81, 1.01) | 0.95 (0.76, 1.17) | 0.98 (0.84, 1.15) |
| **Charlson-Deyo score** | | | | |
| 0 | 1 | 1 | 1 | 1 |
| 1 | 1.03*** (1.01, 1.04) | 1.05*** (1.03, 1.07) | 1.01 (0.98, 1.04) | 1.00 (0.97, 1.02) |
| 2 | 1.04** (1.01, 1.07) | 1.05** (1.02, 1.09) | 1.03 (0.97, 1.11) | 1.05* (1.01, 1.09) |
| 3 | 1.12*** (1.08, 1.16) | 1.09*** (1.04, 1.14) | 1.10 (0.98, 1.25) | 1.05 (0.99, 1.12) |
| **Tumor grade** | | | | |
| I | 1 | 1 | 1 | 1 |
| II | 1.03*** (1.02, 1.05) | 1.03*** (1.02, 1.05) | 1.03 (1.00, 1.06) | 1.04*** (1.02, 1.06) |
| III | 0.99* (0.97, 1.00) | 1.00 (0.98, 1.02) | 0.98 (0.94, 1.01) | 1.01 (0.98, 1.03) |
| IV | 1.06 (0.99, 1.14) | 1.14* (1.02, 1.27) | 0.98 (0.81, 1.18) | 0.94 (0.83, 1.07) |
| **Year of diagnosis** | 1.02*** (1.02, 1.03) | 1.02*** (1.02, 1.02) | 1.03*** (1.03, 1.03) | 1.03*** (1.02, 1.03) |

Significance: * < 0.05, ** < 0.01, *** < 0.001

**Table S12**: Estimated hazard ratios with 95% confidence intervals from adjusted Cox models stratified by living area

| **Characteristics** | **Hazard ratio with 95% confidence interval** | | |
| --- | --- | --- | --- |
|  | **Subset model: Metro (n = 240,968)** | **Subset model: Urban (n = 31,432 )** | **Subset model: Rural (n = 3,952)** |
| **Primary treatment** | | | |
| Surgery | 1 | 1 | 1 |
| Chemotherapy | 1.45*** (1.35, 1.57) | 1.17* (1, 1.38) | 1.22 (0.76, 1.95) |
| Radiation | 1.45*** (1.22, 1.71) | 1.44 (0.99, 2.1) | 1.09 (0.24, 5.07) |
| Endocrine therapy | 1.39*** (1.25, 1.54) | 1.43** (1.12, 1.83) | 1.31 (0.73, 2.32) |
| **Great circle distance in miles** | | | |
| < 10.0 | 1 | 1 | 1 |
| 10.1-20.0 | 0.95 (0.89, 1.02) | 1.00 (0.8, 1.24) | 0.76 (0.4, 1.43) |
| > 20.0 | 0.88** (0.81, 0.96) | 0.82 (0.60, 1.12) | 0.89 (0.44, 1.77) |
| **Weeks from diagnosis to initial treatment** | | | |
| < 8 | 1 | 1 | 1 |
| 8 - 12 | 1.05 (0.98, 1.12) | 1.01 (0.88, 1.17) | 0.86 (0.52, 1.4) |
| > 12 | 1.25*** (1.13, 1.37) | 1.29* (1.05, 1.57) | 1.24 (0.67, 2.28) |
| **Race and ethnicity** | | | |
| White, Non-Hispanic | 1 | 1 | 1 |
| Black, Non-Hispanic | 0.96 (0.87, 1.06) | 0.98 (0.75, 1.27) | 0.88 (0.49, 1.58) |
| Hispanic | 0.77* (0.62, 0.96) | 0.79 (0.31, 2.03) | - |
| Asian, Non-Hispanic | 0.61*** (0.52, 0.72) | 1.05 (0.52, 2.12) | - |
| AIAN, Non-Hispanic | 1.46* (1.03, 2.05) | 0.98 (0.57, 1.71) | 1.49 (0.55, 4.02) |
| NHPI, Non-Hispanic | 1.38* (1.01, 1.88) | 2.29*** (1.49, 3.52) | - |
| Other | 0.78 (0.57, 1.06) | 0.41 (0.09, 1.77) | 0.82 (0.22, 3.11) |
| **Age at diagnosis** | | | |
| < 50 | 1 | 1 | 1 |
| 50-59 | 1.35*** (1.25, 1.47) | 1.54*** (1.27, 1.88) | 1.91 (0.92, 3.95) |
| 60-69 | 2.07*** (1.89, 2.26) | 1.91*** (1.58, 2.31) | 3.08** (1.5, 6.32) |
| 70-79 | 4.24*** (3.78, 4.75) | 3.64*** (2.92, 4.54) | 3.95*** (1.76, 8.88) |
| 80+ | 11.74*** (10.36, 13.3) | 9.86*** (7.69, 12.65) | 10.68*** (4.65, 24.5) |
| **Education: percent no high school degree** | | | |
| No HSD >=15.3% | 1 | 1 | 1 |
| No HSD 9.1% - 15.2% | 1.09 (0.96, 1.24) | 0.87 (0.73, 1.02) | 0.69* (0.49, 0.99) |
| No HSD 5.0% - 9.0% | 0.97 (0.84, 1.12) | 0.94 (0.72, 1.23) | 0.62* (0.4, 0.99) |
| No HSD < 5.0% | 0.91 (0.79, 1.06) | 0.76 (0.57, 1.03) | 0.50* (0.26, 0.94) |
| **Median household income** | | | |
| < $46,227 | 1 | 1 | 1 |
| $46,227 - $57,856 | 0.90 (0.78, 1.04) | 0.92 (0.78, 1.08) | 1.06 (0.74, 1.53) |
| $57,857 - $74,062 | 0.84* (0.72, 0.97) | 0.71** (0.58, 0.88) | 0.85 (0.53, 1.38) |
| >= $74,063 | 0.76** (0.64, 0.9) | 0.82 (0.58, 1.16) | 2.00 (0.94, 4.26) |
| **Insurance status** | | | |
| Private | 1 | 1 | 1 |
| Uninsured | 1.48*** (1.24, 1.77) | 2.39*** (1.67, 3.44) | 1.23 (0.3, 5.01) |
| Medicaid | 1.66*** (1.51, 1.84) | 1.69*** (1.36, 2.09) | 3.10*** (1.69, 5.67) |
| Medicare | 1.39*** (1.29, 1.51) | 1.63*** (1.42, 1.88) | 1.72** (1.15, 2.57) |
| Other Government | 1.29* (1.04, 1.6) | 1.22 (0.76, 1.96) | 0.39 (0.06, 2.43) |
| **Facility type** | | | |
| Comprehensive Community Cancer Program | 1 | 1 | 1 |
| Academic/Research Program | 0.93 (0.74, 1.17) | 0.88 (0.71, 1.08) | 0.76 (0.52, 1.12) |
| Community Cancer Program | 1.44** (1.16, 1.79) | 1.12 (0.83, 1.51) | 0.81 (0.55, 1.2) |
| Integrated Network Cancer Program | 1.15 (0.91, 1.46) | 1.32 (0.88, 1.96) | 1.10 (0.72, 1.68) |
| **Facility location** | | | |
| West | 1 | 1 | 1 |
| South | 0.94 (0.71, 1.25) | 0.98 (0.69, 1.39) | 1.29 (0.69, 2.39) |
| Midwest | 0.90 (0.67, 1.21) | 1.00 (0.73, 1.37) | 0.95 (0.54, 1.67) |
| Northeast | 1.08 (0.78, 1.5) | 1.03 (0.71, 1.49) | 1.07 (0.57, 2) |
| **Charlson-Deyo score** | | | |
| 0 | 1 | 1 | 1 |
| 1 | 1.50*** (1.41, 1.6) | 1.41*** (1.24, 1.6) | 1.51** (1.11, 2.05) |
| 2 | 1.97*** (1.77, 2.19) | 2.20*** (1.79, 2.7) | 2.61*** (1.58, 4.32) |
| 3 | 2.81*** (2.42, 3.26) | 3.53*** (2.7, 4.62) | 1.85 (0.96, 3.56) |
| **Cancer stage** | | | |
| 0 | 1 | 1 | 1 |
| I | 1.26*** (1.18, 1.33) | 1.31*** (1.12, 1.53) | 1.26 (0.79, 1.99) |
| II | 1.78*** (1.67, 1.9) | 1.75*** (1.5, 2.03) | 2.12** (1.33, 3.39) |
| III | 3.09*** (2.83, 3.37) | 3.22*** (2.6, 3.98) | 2.55** (1.43, 4.54) |
| IV | 10.14*** (9.02, 11.41) | 10.87*** (8.24, 14.33) | 7.86*** (3.75, 16.49) |
| **Tumor grade** | | | |
| I | 1 | 1 | 1 |
| II | 1.11*** (1.05, 1.17) | 1.18* (1.04, 1.34) | 1.11 (0.81, 1.52) |
| III | 1.27*** (1.2, 1.35) | 1.29*** (1.12, 1.48) | 1.22 (0.82, 1.82) |
| IV | 1.26 (0.97, 1.64) | 1.29 (0.67, 2.49) | 2.63 (0.54, 12.68) |
| **Year of diagnosis** | 1.33*** (1.2, 1.48) | 1.36*** (1.2, 1.53) | 1.48** (1.15, 1.89) |

Significance: * < 0.05, ** < 0.01, *** < 0.001

**-** : not estimable due to small sample size
--: infinite estimated hazard ratio

**Table S13**: Estimated hazard ratios with 95% confidence intervals from adjusted Cox models stratified by facility type

| **Characteristics** | **Hazard ratio with 95% confidence interval** | | | |
| --- | --- | --- | --- | --- |
|  | **Comprehensive Community Cancer Program (n = 121,650)** | **Academic/Research Program (n = 90,811)** | **Community Cancer Program (n = 20,677)** | **Integrated Network Cancer Program (n = 50,028)** |
| **Primary treatment** | | | | |
| Surgery | 1 | 1 | 1 | 1 |
| Chemotherapy | 1.42*** (1.28, 1.58) | 1.44*** (1.27, 1.63) | 1.41*** (1.17, 1.7) | 1.34*** (1.15, 1.55) |
| Radiation | 1.59*** (1.21, 2.09) | 1.31* (1.04, 1.66) | 1.29 (0.76, 2.19) | 1.48* (1.09, 2.02) |
| Endocrine therapy | 1.40*** (1.21, 1.62) | 1.53*** (1.28, 1.81) | 1.18 (0.89, 1.57) | 1.25* (1.03, 1.51) |
| **Great circle distance in miles** | | | | |
| < 10.0 | 1 | 1 | 1 | 1 |
| 10.1-20.0 | 0.99 (0.89, 1.11) | 0.94 (0.85, 1.05) | 0.87* (0.75, 0.99) | 0.97 (0.86, 1.09) |
| > 20.0 | 0.93 (0.84, 1.04) | 0.86* (0.75, 0.99) | 0.84 (0.69, 1.03) | 0.77 (0.58, 1.01) |
| **Weeks from diagnosis to initial treatment** | | | | |
| < 8 | 1 | 1 | 1 | 1 |
| 8 - 12 | 1.02 (0.93, 1.11) | 1.04 (0.93, 1.16) | 1.20* (1.02, 1.41) | 1.04 (0.92, 1.19) |
| > 12 | 1.23** (1.09, 1.40) | 1.27** (1.09, 1.47) | 1.36* (1.01, 1.82) | 1.19 (0.99, 1.44) |
| **Race and ethnicity** | | | | |
| White, Non-Hispanic | 1 | 1 | 1 | 1 |
| Black, Non-Hispanic | 0.98 (0.83, 1.16) | 0.99 (0.85, 1.16) | 1.07 (0.82, 1.39) | 0.79* (0.66, 0.95) |
| Hispanic | 0.91 (0.7, 1.18) | 0.58** (0.38, 0.88) | 1.28 (0.87, 1.87) | 0.68 (0.44, 1.07) |
| Asian, Non-Hispanic | 0.72** (0.58, 0.91) | 0.64*** (0.53, 0.79) | 0.80 (0.5, 1.28) | 0.40*** (0.26, 0.61) |
| AIAN, Non-Hispanic | 1.28 (0.9, 1.83) | 1.05 (0.59, 1.86) | 2.44* (1.04, 5.73) | 0.66 (0.22, 1.97) |
| NHPI, Non-Hispanic | 1.33 (0.73, 2.43) | 1.36 (0.95, 1.94) | 1.94* (1.11, 3.38) | 2.56** (1.37, 4.79) |
| Other | 0.83 (0.53, 1.31) | 0.76 (0.46, 1.25) | 0.31 (0.07, 1.32) | 0.83 (0.5, 1.39) |
| **Education: percent no high school degree** | | | | |
| No HSD >=15.3% | 1 | 1 | 1 | 1 |
| No HSD 9.1% - 15.2% | 1.02 (0.86, 1.2) | 1.04 (0.85, 1.29) | 1.12 (0.9, 1.39) | 0.97 (0.79, 1.18) |
| No HSD 5.0% - 9.0% | 0.89 (0.74, 1.07) | 1.03 (0.8, 1.34) | 0.94 (0.71, 1.23) | 0.93 (0.74, 1.17) |
| No HSD < 5.0% | 0.80** (0.67, 0.94) | 0.88 (0.64, 1.21) | 1.22 (0.86, 1.74) | 0.84 (0.65, 1.1) |
| **Median household income** | | | | |
| < $46,227 | 1 | 1 | 1 | 1 |
| $46,227 - $57,856 | 0.77** (0.63, 0.94) | 1.09 (0.95, 1.24) | 0.91 (0.72, 1.15) | 1.04 (0.85, 1.26) |
| $57,857 - $74,062 | 0.75* (0.6, 0.94) | 0.91 (0.76, 1.1) | 0.78 (0.6, 1.01) | 0.92 (0.75, 1.12) |
| >= $74,063 | 0.77* (0.61, 0.97) | 0.83 (0.63, 1.09) | 0.80 (0.59, 1.08) | 0.74* (0.57, 0.95) |
| **Insurance status** | | | | |
| Private | 1 | 1 | 1 | 1 |
| Uninsured | 1.61*** (1.24, 2.09) | 1.50*** (1.2, 1.87) | 1.36 (0.88, 2.11) | 1.74** (1.17, 2.59) |
| Medicaid | 1.84*** (1.6, 2.12) | 1.51*** (1.28, 1.79) | 1.55** (1.19, 2.02) | 1.88*** (1.55, 2.28) |
| Medicare | 1.51*** (1.39, 1.65) | 1.29*** (1.13, 1.48) | 1.59*** (1.35, 1.87) | 1.44*** (1.28, 1.62) |
| Other Government | 1.23 (0.92, 1.64) | 0.98 (0.65, 1.47) | 1.31 (0.78, 2.22) | 1.41 (0.88, 2.26) |
| **Living area** | | | | |
| Metro | 1 | 1 | 1 | 1 |
| Urban | 1.02 (0.87, 1.19) | 1.00 (0.84, 1.19) | 0.98 (0.78, 1.24) | 1.19 (0.73, 1.93) |
| Rural | 1.22 (0.9, 1.64) | 0.97 (0.73, 1.28) | 0.82 (0.59, 1.15) | 1.35 (0.88, 2.07) |
| **Facility location** | | | | |
| West | 1 | 1 | 1 | 1 |
| South | 1.13 (0.87, 1.47) | 1.27 (0.95, 1.7) | 0.91 (0.59, 1.41) | 0.44** (0.23, 0.82) |
| Midwest | 1.07 (0.83, 1.38) | 1.29* (1.01, 1.65) | 0.88 (0.58, 1.36) | 0.42** (0.23, 0.79) |
| Northeast | 1.18 (0.82, 1.71) | 1.45 (0.89, 2.35) | 1.38 (0.8, 2.38) | 0.41** (0.21, 0.77) |
| **Charlson-Deyo score** | | | | |
| 0 | 1 | 1 | 1 | 1 |
| 1 | 1.49*** (1.37, 1.62) | 1.47*** (1.3, 1.67) | 1.41*** (1.21, 1.65) | 1.51*** (1.36, 1.67) |
| 2 | 1.93*** (1.69, 2.21) | 2.07*** (1.68, 2.54) | 1.87*** (1.48, 2.36) | 2.15*** (1.79, 2.57) |
| 3 | 2.80*** (2.37, 3.31) | 3.23*** (2.38, 4.38) | 2.33*** (1.69, 3.2) | 2.90*** (2.24, 3.76) |
| **Cancer stage** | | | | |
| 0 | 1 | 1 | 1 | 1 |
| I | 1.28*** (1.18, 1.4) | 1.29*** (1.15, 1.46) | 1.32*** (1.13, 1.55) | 1.16** (1.04, 1.29) |
| II | 1.84*** (1.67, 2.02) | 1.81*** (1.62, 2.01) | 1.77*** (1.5, 2.1) | 1.67*** (1.46, 1.91) |
| III | 3.25*** (2.83, 3.72) | 2.94*** (2.53, 3.42) | 3.29*** (2.64, 4.1) | 3.05*** (2.58, 3.6) |
| IV | 9.90*** (8.24, 11.9) | 10.53*** (8.6, 12.9) | 10.62*** (7.86, 14.35) | 9.56*** (7.52, 12.15) |
| **Cancer subtype** | | | | |
| Luminal A | 1 | 1 | 1 | 1 |
| Luminal B | 1.37 (0.91, 2.05) | 1.32 (0.8, 2.16) | 1.18 (0.57, 2.44) | 2.38** (1.42, 4) |
| HER2-Enriched | 0.79 (0.38, 1.64) | 1.62 (0.75, 3.46) | 0.45 (0.12, 1.7) | 1.61 (0.61, 4.23) |
| Triple-Negative | 2.11*** (1.36, 3.28) | 1.98* (1.1, 3.57) | 1.18 (0.4, 3.45) | 2.51** (1.3, 4.84) |
| **Tumor grade** | | | | |
| I | 1 | 1 | 1 | 1 |
| II | 1.13*** (1.05, 1.21) | 1.11* (1.02, 1.2) | 1.17* (1, 1.36) | 1.10 (0.99, 1.21) |
| III | 1.29*** (1.18, 1.41) | 1.31*** (1.19, 1.43) | 1.28** (1.08, 1.53) | 1.20** (1.08, 1.34) |
| IV | 1.53** (1.11, 2.11) | 0.91 (0.5, 1.65) | 1.18 (0.49, 2.86) | 0.96 (0.51, 1.83) |
| **Year of diagnosis** | 1.42 (1.24, 1.63) | 1.38* (1.04, 1.84) | 1.28*** (1.12, 1.47) | 1.22** (1.08, 1.37) |
| **Age at diagnosis** | | | | |
| < 50 | 1 | 1 | 1 | 1 |
| 50-59 | 1.38*** (1.24, 1.54) | 1.34*** (1.16, 1.54) | 1.28* (1.01, 1.61) | 1.54*** (1.3, 1.83) |
| 60-69 | 1.91*** (1.71, 2.15) | 2.18*** (1.87, 2.56) | 1.80*** (1.43, 2.26) | 2.23*** (1.89, 2.64) |
| 70-79 | 3.86*** (3.39, 4.4) | 4.53*** (3.59, 5.7) | 3.03*** (2.34, 3.94) | 4.62*** (3.94, 5.41) |
| 80+ | 9.97*** (8.59, 11.58) | 13.26*** (10.28, 17.1) | 8.68*** (6.53, 11.54) | 13.60*** (11.51, 16.06) |

Significance: * < 0.05, ** < 0.01, *** < 0.001

**Figure S1**: Predicted survival probability differences by timeliness of care categories from the adjusted Cox model for the overall population


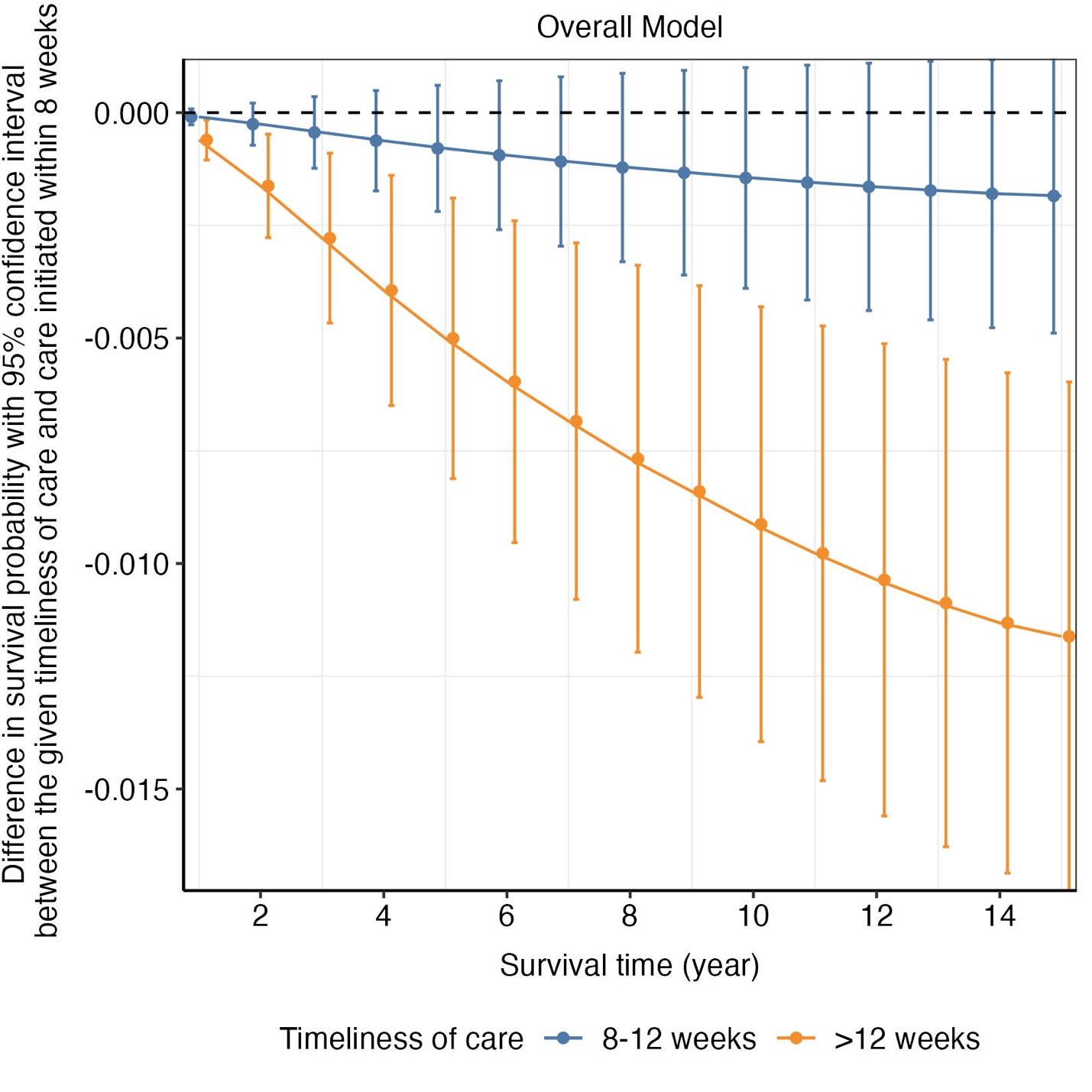


**Legend**: The plot shows predicted survival probability differences with 95% confidence intervals between timeliness of care categories (8–12 weeks [blue bar] and >12 weeks [orange bar]) and timeliness of care within 8 weeks, from adjusted Cox models for the overall population

**Figure S2**: Predicted survival probability differences by timeliness of care categories from adjusted Cox models for the cancer stage (stage 0, I, II, III, IV)

**
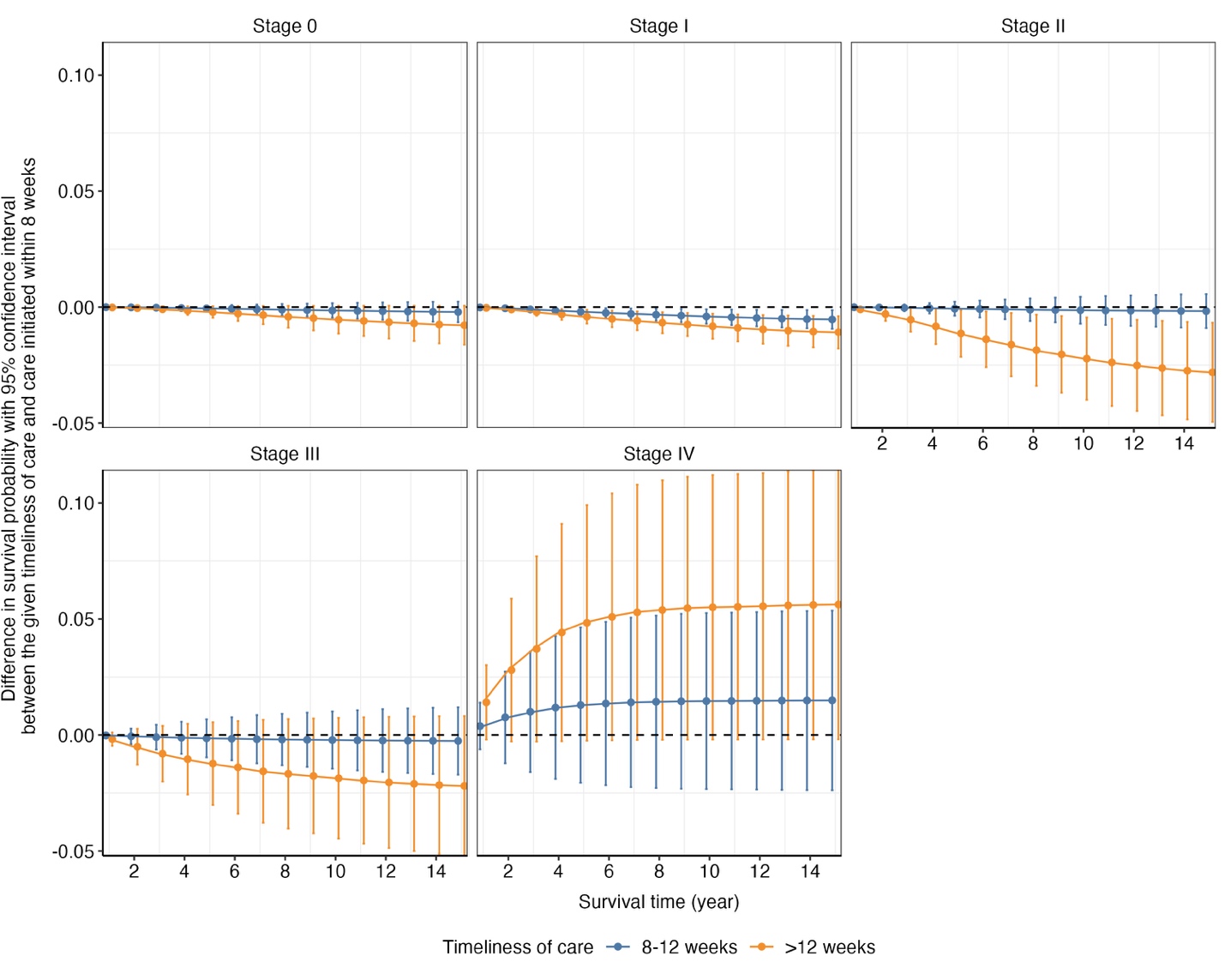
**

**Legend**: Plots from top left to bottom right show predicted survival probability differences with 95% confidence intervals between timeliness of care categories (8–12 weeks [blue bar] and >12 weeks [orange bar]) and timeliness of care within 8 weeks, from adjusted Cox models for the cancer stage (stage 0, I, II, III, IV)

**Figure S3**: Predicted survival probability differences by timeliness of care categories from adjusted Cox models for the molecular subtype (luminal A, luminal B, HER2-Enriched, triple-negative)

**
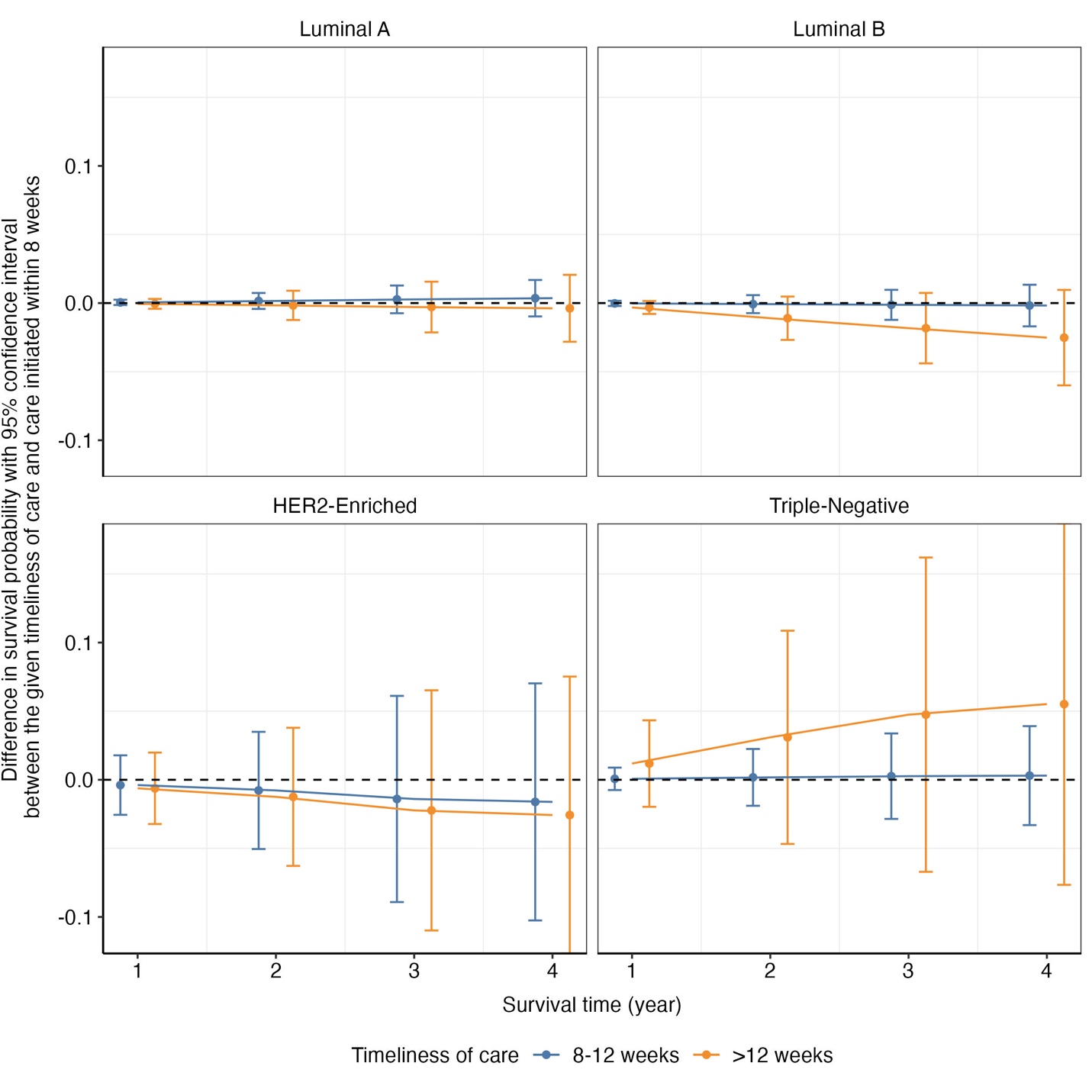
**

**Legend**: Plots from top left to bottom right show predicted survival probability differences with 95% confidence intervals between timeliness of care categories (8–12 weeks [blue bar] and >12 weeks [orange bar]) and timeliness of care within 8 weeks, from adjusted Cox models for the molecular subtype (luminal A, luminal B, HER2-Enriched, triple-negative)

**Figure S4**: Predicted survival probability differences by timeliness of care categories from adjusted Cox models for the primary modality (surgery, chemotherapy, radiation, endocrine therapy)

**
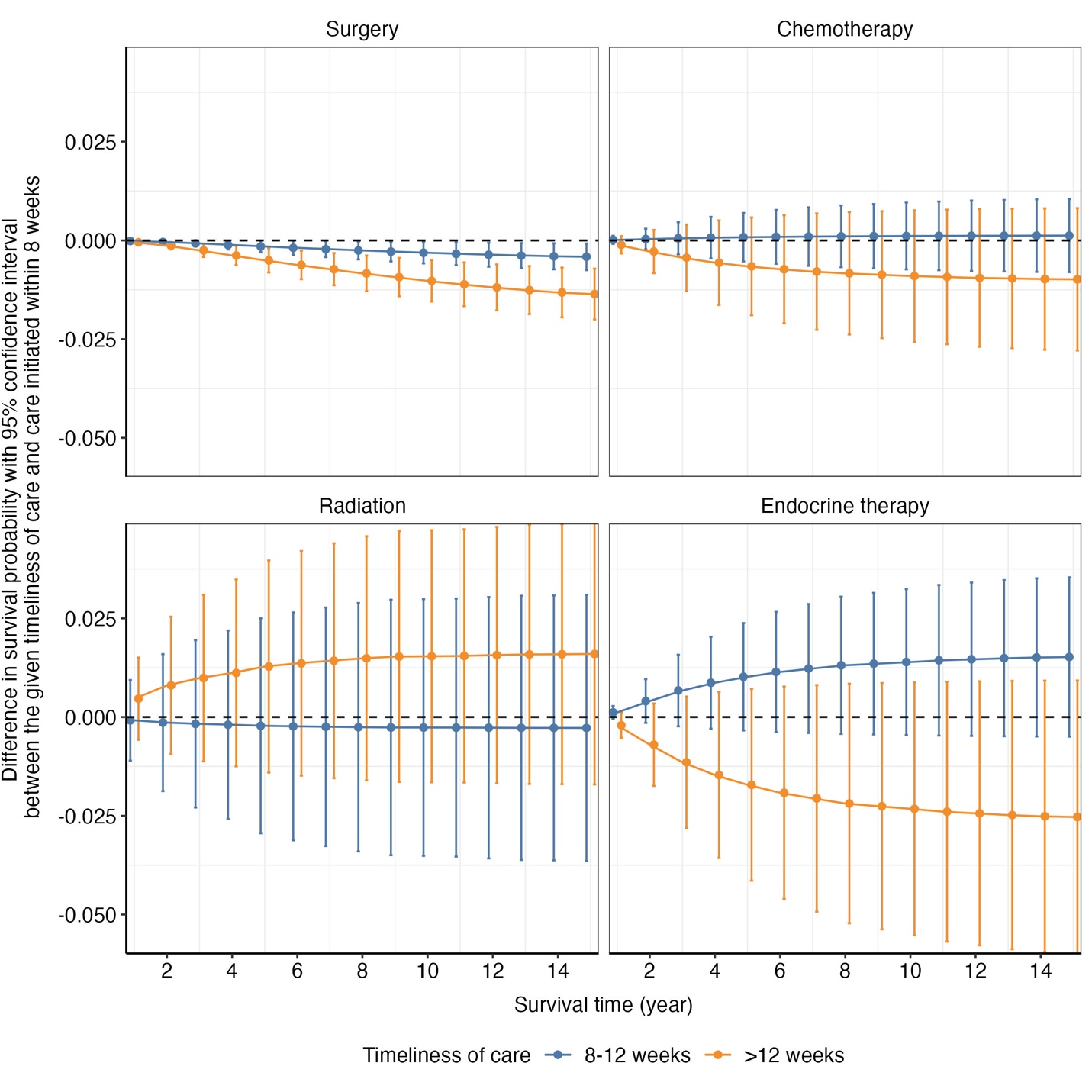
**

**Legend**: Plots from top left to bottom right show predicted survival probability differences with 95% confidence intervals between timeliness of care categories (8–12 weeks [blue bar] and >12 weeks [orange bar]) and timeliness of care within 8 weeks, from adjusted Cox models for the primary modality (surgery, chemotherapy, radiation, endocrine therapy)

**Figure S5**: Predicted survival probability differences by timeliness of care categories from adjusted Cox models for the living area (metro, urban, rural)

**
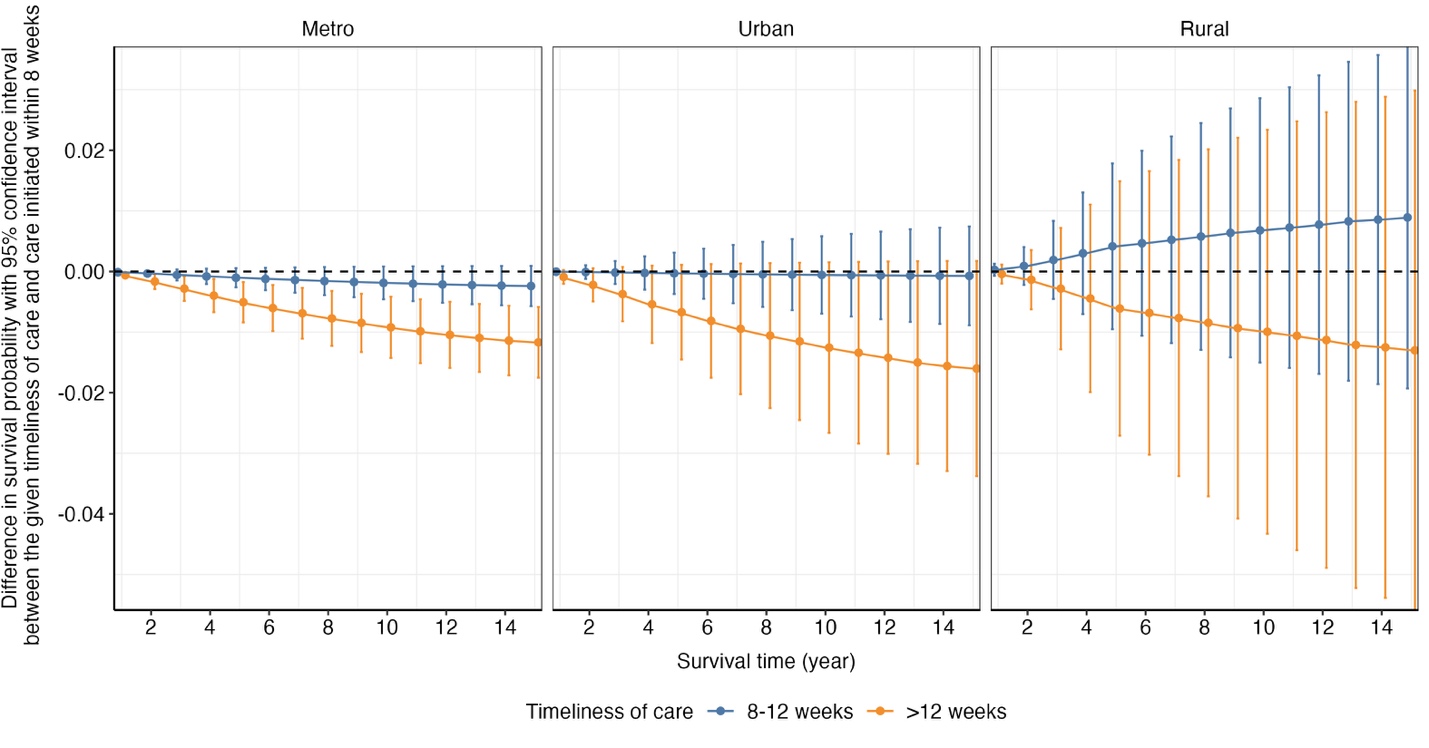
**

**Legend**: Plots from top left to bottom right show predicted survival probability differences with 95% confidence intervals between timeliness of care categories (8–12 weeks [blue bar] and >12 weeks [orange bar]) and timeliness of care within 8 weeks, from adjusted Cox models for the living area (metro, urban, rural)

**Figure S6**: Predicted survival probability differences by timeliness of care categories from adjusted Cox models for the facility type (comprehensive community cancer program, academic/research program, community cancer program, integrated network cancer program)

**
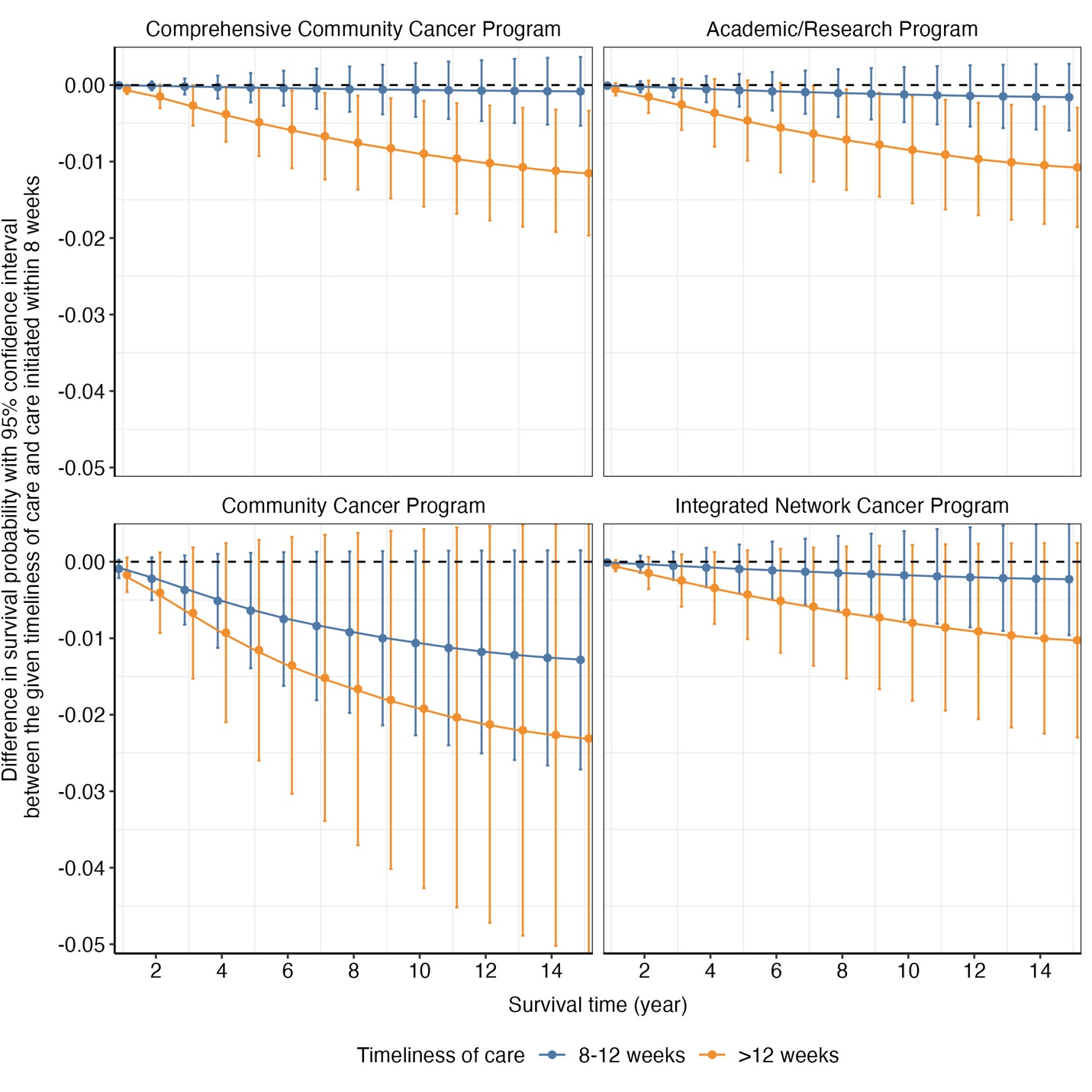
**

**Legend**: Plots from top left to bottom right show predicted survival probability differences with 95% confidence intervals between timeliness of care categories (8–12 weeks [blue bar] and >12 weeks [orange bar]) and timeliness of care within 8 weeks, from adjusted Cox models for the facility type (comprehensive community cancer program, academic/research program, community cancer program, integrated network cancer program)
